# Cancer-associated fibroblasts are associated with neo-adjuvant treatment response in oesophageal adenocarcinoma

**DOI:** 10.1038/s41416-025-03080-8

**Published:** 2025-07-10

**Authors:** Robert C. Walker, Stella P. Breininger, Benjamin P. Sharpe, Jack Harrington, Ian Reddin, Carmen Tse, Rushda Rajak, Annette Hayden, Saqib Rahman, Ben Grace, Fereshteh Izadi, Jonathan West, The OCCAMs Consortium, Robert C. Walker, Rebecca C. Fitzgerald, Paul A. W. Edwards, Nicola Grehan, Barbara Nutzinger, Caitriona Hughes, Elwira Fidziukiewicz, Shona MacRae, Alex Northrop, Xiaodun Li, Annalise Katz-Summercorn, Sujath Abbas, Maria O’Donovan, Ahmad Miremadi, Shalini Malhotra, Monika Tripathi, EC Smyth, Simon Tavaré, Andy G. Lynch, Matthew Eldridge, Maria Secrier, Ginny Devonshire, Sriganesh Jammula, Aisling M. Redmond, Sarah Killcoyne, Amber Grantham, Adrienn Blasco, Jim Davies, Charles Crichton, Nick Carroll, Peter Safranek, Andrew Hindmarsh, Vijayendran Sujendran, J. Robert O’Neill, Stephen J. Hayes, Yeng Ang, Andrew Sharrocks, Shaun R. Preston, Sarah Oakes, Izhar Bagwan, Vicki Save, Richard J. E. Skipworth, Ted R. Hupp, Olga Tucker, Andrew Beggs, Philippe Taniere, Sonia Puig, Gianmarco Contino, Ben L. Grace, Hugh Barr, Neil Shepherd, Oliver Old, Jesper Lagergren, James Gossage, Andrew Davies, Fuju Chang, Janine Zylstra, Ula Mahadeva, Vicky Goh, Francesca D. Ciccarelli, Grant Sanders, Richard Berrisford, Catherine Harden, Mike Lewis, Ed Cheong, Bhaskar Kumar, Simon L. Parsons, Irshad Soomro, Philip Kaye, John Saunders, Laurence Lovat, Rehan Haidry, Michael Scott, Sharmila Sothi, Sari Suortamo, Suzy Lishman, George B. Hanna, Christopher J. Peters, Krishna Moorthy, Anna Grabowska, Richard Turkington, Damian McManus, Helen Coleman, David Khoo, Will Fickling, Tom D. L. Crosby, Russell D. Petty, Timothy J. Underwood, Maria Secrier, Zoë S. Walters, Matthew J. J. Rose-Zerilli, Timothy J. Underwood

**Affiliations:** 1 School of Cancer Sciences, Faculty of Medicine, University of Southampton, Southampton, UK; 2 Department of Pathology, University Hospital Southampton NHS Foundation Trust, Southampton, UK; 3 Institute for Life Science, University of Southampton, Southampton, UK; 4 UCL Genetics Institute, University College London, London, UK; 5 Medical Research Council Cancer Unit, Hutchison/Medical Research Council Research Centre, University of Cambridge, Cambridge, UK; 6 Cancer Research UK Cambridge Institute, University of Cambridge, Cambridge, UK; 7 Department of Histopathology, Addenbrooke’s Hospital, Cambridge, UK; 8 Department of Oncology, Cambridge University Hospitals NHS Foundation Trust, Cambridge, UK; 9 Department of Computer Science, University of Oxford, Oxford, UK; 10 Cambridge University Hospitals NHS Foundation Trust, Cambridge, UK; 11 Salford Royal NHS Foundation Trust, Salford, M6 8HD UK; 12 Faculty of Medical and Human Sciences, University of Manchester, Manchester, UK; 13 Wigan and Leigh NHS Foundation Trust Wigan, Manchester, UK; 14 GI Science Centre, University of Manchester, Manchester, UK; 15 Royal Surrey County Hospital NHS Foundation Trust, Guildford, UK; 16 Edinburgh Royal Infirmary, Edinburgh, UK; 17 Edinburgh University, Edinburgh, UK; 18 University Hospitals Birmingham NHS Foundation Trust, Birmingham, UK; 19 Heart of England NHS Foundation Trust, Birmingham, UK; 20 Institute of Cancer and Genomic Sciences, University of Birmingham, Birmingham, UK; 21 Gloucester Royal Hospital, Gloucester, UK; 22 Guy’s and St Thomas’s NHS Foundation Trust, London, UK; 23 Karolinska Institute, Stockholm, Sweden; 24 King’s College London, London, UK; 25 Plymouth Hospitals NHS Trust, Plymouth, UK; 26 Norfolk and Norwich University Hospital NHS Foundation Trust, Norwich, UK; 27 Nottingham University Hospitals NHS Trust, Nottingham, UK; 28 University College London, London, UK; 29 Wythenshawe Hospital, Manchester, UK; 30 University Hospitals Coventry and Warwickshire NHS Trust, Coventry, UK; 31 Peterborough City Hospital, Peterborough Hospitals NHS Trust, Peterborough, UK; 32 Department of Surgery and Cancer, Imperial College, London, UK; 33 Queen’s Medical Centre, University of Nottingham, Nottingham, UK; 34 Centre for Cancer Research and Cell Biology, Queen’s University Belfast, Belfast, NI UK; 35 Queen’s Hospital, Romford, UK; 36 Velindre University NHS Trust, Cardiff, Wales UK; 37 Medical Oncology, Division of Molecular and Clinical Medicine, Ninewells Hospital and Medical School, University of Dundee, Dundee, Scotland

**Keywords:** Oesophageal cancer, Oesophageal cancer

## Abstract

**Background:**

Neoadjuvant treatment (NAT) in oesophageal adenocarcinoma (EAC) is characterised by differential responses between patients and treatment modalities. The components of the tumour microenvironment (TME) that contribute to this are unknown. We explored this, focusing on cancer-associated fibroblasts (CAF) an abundant TME component.

**Methods:**

We performed histopathologic, single-cell RNA sequencing and transcriptomic analysis on 26 patients, stratified by pathological response to NAT, and validated a prognostic model in genomic consortia cohorts. Patient-derived cells were used to model CAF phenotypes in vitro.

**Results:**

We observed changes in the TME in response to the NAT received. Specific changes in fibroblasts correlated with treatment response and altered gene expression associated with NAT type. Three myofibroblastic phenotypes dominate the TME, two of which persist in non-responders and could only be partially re-capitulated in vitro using co-culture with cancer cells or TGF-β. A two-gene NAT fibrotic signature was an independent prognostic indicator in chemo/chemoradiotherapy treated patients (HR = 2.47, *p* = 0.029).

**Conclusions:**

This study provides a compendium of cell phenotypes in EAC across the current NAT treatment pathway that provides insights into CAF biology and cancer progression. MyoCAFs represent an axis to repurpose agents to enhance current therapies and immunotherapy.

## Introduction

Oesophageal adenocarcinoma (EAC) is resistant to current therapies and precision treatments have been disappointing, hampered by a scarcity of recurrent, targetable driver events [1] and cellular heterogeneity within and between primary tumours and metastases [2]. Clinical controversy exists in the curative setting regarding the best neoadjuvant treatment with some favouring neoadjuvant chemoradiotherapy (CRT) and others preferring peri-operative chemotherapy (CT). The most recent randomised clinical trial which compared these two modalities head-to-head demonstrated superiority for CT over CRT for overall and disease-free survival, but there may still be circumstances where CRT will be preferred, especially if the circumferential surgical margin is threatened [3]. Studies consistently demonstrate improved pathological response rates with CRT, but this does not translate to a survival advantage. When EAC recurs after treatment given with curative intent, this is most often at distant sites (e.g., liver, lung, bone), meaning that many clinicians favour perioperative CT, especially for tumours of the gastro-oesophageal junction and those with lymph node positive (N+) disease. Relatively little is known about the effects of neoadjuvant treatments on noncancer cells of the tumour microenvironment in EAC, and it is likely that the combination of direct on-target effects of chemotherapy/CRT on cancer cells and the effects of these treatments on the TME in general determine outcome and may explain the differential clinical outcomes overserved in practice.

In this obfuscated treatment landscape, the introduction of immunotherapy for EAC is difficult. Adjuvant nivolumab is indicated after neoadjuvant CRT and surgery where cancer remains at the time of resection [4], but the role of immune checkpoint blockade in neoadjuvant or perioperative practice is not proven. Early data from large phase 3 trials suggest improvements in pathological complete response (pCR), but are yet to demonstrate a commensurate benefit in overall survival [5, 6], and these are mainly trials of gastric and junctional cancers, not cancers of the oesophagus. It is almost certain that the introduction of immunotherapy for oesophageal cancer will be in limited biomarker-selected sub-groups and a far greater understanding of the EAC tumour microenvironment (TME) is required to understand the response to existing treatments (CT & CRT) and how this might prime for immune checkpoint blockade (ICB).

Many cell types of the tumour microenvironment (TME) have been shown to be important determinants of cancer treatment and prognosis. Cancer-associated fibroblasts (CAFs) are recognised across cancers and are prognostic in EAC [7]. We have shown that the tumour-promoting and chemoprotective properties of CAFs are associated with an activated myofibroblast (α-Smooth Muscle Actin/*ACTA2*^+^, Periostin/POSTN^+^) phenotype that can be reversed with phosphodiesterase type 5 inhibitors (PDE5i) [8]. CAFs have a proven role in augmenting the tumour immune response. Very little is known about CAF heterogeneity in EAC, how the EAC ecosystem changes after neoadjuvant treatment and how this might determine current clinical outcomes, the response to immunotherapy or other emerging therapies [9, 10]. To provide a framework for understanding the functional and clinical relevance of CAF heterogeneity in the EAC TME, we dissected their role in treatment response by performing single cell RNA sequencing (scRNA-seq) on resected tissue following neoadjuvant treatment.

## Methods

### Patient cohort

Twenty-six patients with EAC undergoing treatment with curative intent were recruited. Samples of their tumour were taken at either a staging investigation (*n* = 4) or resection (*n* = 22). Samples from staging investigation consisted of six to eight 2 × 2 mm endoscopic biopsies and samples from resection consisted of a single 8 mm punch biopsy.

### Ethics approval and consent to participate

Human tissue samples were obtained from patients through University Hospitals Southampton NHS trust after informed consent (Newcastle & North Tyneside 1 Research Ethics Committee, REC No: 18/NE/0234). All methods were performed in accordance with the relevant guidelines and regulations.

### Immunohistochemistry

Immunohistochemical staining was performed on 4 μm thick formalin-fixed paraffin embedded tissue sections. Appropriate heat-induced epitope retrieval for each stain was performed on a Dako PT Link instrument. Staining was performed individually for CD3 (IS503, Dako), EpCAM (M352501-2, Dako), POSTN (ab14041, abcam), α-SMA (M085129-2, Dako), GSN (12953S, Cell Signaling Technologies) and TRPA1 (sc-376495, Santa Cruz Biotechnology) using a Dako Autostainer 48S machine according to the manufacturer’s instructions. A separate adjacent section was taken to perform routine hematoxylin&eosin staining to identify regions containing tumour and normal oesophageal and gastric epithelium.

### Drop-seq

Tissue was promptly disaggregated to a single cell suspension [11] and analysed using Drop-seq [12]. Microfluidics for cell and bead co-encapsulation in droplets used microfluidic devices available from Macosko, with open instrumentation syringe pumps and a short exposure microscope assembled according to instructions on the dropletkitchen repository (https://dropletkitchen.github.io/). 1000 STAMPs per sample were then selected for PCR amplification (15 cycles), library preparation (Nextera XT, Illumina) and Illumina sequencing using a custom read 1 primer on the NextSeq-500 platform. Raw reads were processed and counted using Drop-seq-tools-v2.1, details provided in the Online supplement.

### Unsupervised clustering and downstream analysis

A Seurat (R package version 4.1.1) object was filtered for cell barcodes with 150–4000 genes detected, less than 25% mitochondrial genes and less than 10% threshold for tissue dissociation signature genes. Counts were normalised and variable genes selected using SCTransform with latent variables to control for technical variation, prior to PCA, community detection (Louvain) and visualisation using the RunUMAP function. Cluster-specific genes were detected using the Wilcoxon rank sum test (*p* < 1 × 10^−3^ was considered significant). Cell identity for each cluster was defined by the expression of canonical markers and a priori knowledge (Supplementary Data). Treatment-naïve sample data were used as reference atlas for subsequent automated cell identification in the chemotherapy and CRT-treated datasets using canonical correlation analysis, cells with a prediction score <0.8 were excluded from further analysis. After manual inspection of lineage markers of each dataset, further low-quality or doublet cells were removed, as well as patients with low cellularity (*n* = 5). Module scoring was applied to the cancer cells using The MSigDB Hallmark collection and Seurat’s “AddModuleScore” function. Quiescent cells were not analysed further due to the low number of cells present (*n* = 256). For each separate cell lineage dataset, we iteratively pruned cells where we were uncertain of their cell identity (by marker log-normalised expression <1). The pruned datasets were then re-clustered using the steps described above prior and as described in the manuscript.

### Cell culture and analysis

Human Fetal Foreskin Fibroblast 2 (HFFF2) cells (ECACC Cat-no: 86031405) were maintained in Dulbecco’s modified Eagle’s medium (DMEM, Invitrogen) supplemented with 10% (v/v) foetal calf serum (Autogen Bioclear), 2 mM L-glutamine and 1% penicillin/streptomycin (Invitrogen) at 37 °C and 5% CO_2_. TGF-β was used to induce myofibroblast differentiation and was manufactured in-house by Dr Patrick Duriez. HFFF2 were grown under cell culture conditions for 6 h. After the cells adhered, media was then changed to low serum (1%) DMEM for 24 h before the addition of 2 ng/mL TGF-β or vehicle (4 mM HCl, 0.4 mg/mL BSA) and grown for 72 h. cDNA synthesis was performed using the High-Capacity Reverse Transcription Kit (Applied Biosystems). The TaqMan assays to *POSTN, ACTA2, ZEB1, ZEB2, ATF1*, and *GSN* were run in biological triplicate on the Real-time PCR system (ThermoScientific) with a VIC-TAMRA-labelled β-actin assay (ThermoScientific) as an internal control. Quantities of RNA per well were interpolated from a standard curve, normalised to the internal control samples as indicated. Protein expression was carried out by Western Blotting in biological triplicates. Protein was extracted using cell lysis buffer (Cell Signaling) and resolved on 3–8% Bis-Tris acrylamide gels and transferred onto PVDF membranes using the X-Cell II and iBlot systems (Invitrogen). Blocking and antibody incubations were done in 3% low-fat milk in PBS-0.025% Tween 20, and washes were in PBS–0.1% Tween 20. Images were collected using a CCD camera.

Full methodology is available in the supplementary data.

### Statistical analyses

Statistical analysis was performed using R version 4.1.1. Enrichment of cell types and subtypes was calculated using 2 × 2 contingency table *X*^2^ tests. Categorical variables were compared using the Chi-Squared test. A two-tailed Student’s *t* test was used to analyse parametric data and non-parametric data was analysed by a Mann-Whitney test (unpaired data) or Wilcoxon test (paired data). To assess survival differences, Kaplan-Meier curves were produced and analysed by Log-rank testing. *p* values < 0.05 were regarded as statistically significant. Unless stated otherwise, *p* values are represented as follows, *p < 0.05, **p < 0.01, ***p < 0.001 and ****p < 0.0001.

## Results

### Changes to the tumour microenvironment in response to neoadjuvant treatment

**Fig. 1:**
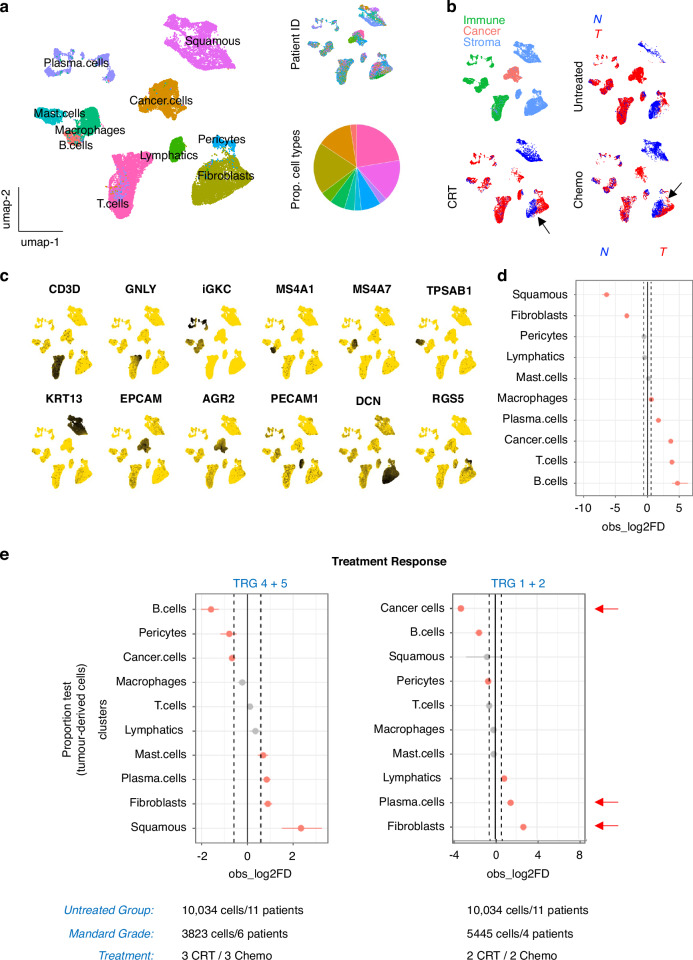
Treatment response and the EAC TME. **a** UMAP of cell lineages. Inset: UMAP of patient ID and a Pie chart of cell type proportions. **b** UMAP of tumour microenvironment components and cell source (T = tumour (red dots), N= normal (blue dots)) stratified by treatment status. Black arrows indicate the cancer-associated fibroblast cell clusters. **c** Feature Plots of canonical cell lineage marker expression in individual cells (each cell is a dot) where a gold colour indicates a specific transcript is not detected/expressed, and an increasing grey-black scale indicates increases in scaled expression levels. **d** scProportionTest plots of observed log2 fold differences between tumour (T) and normal (N) samples. FDR < 0.05 are indicated by the vertical dotted lines. **e** scProportionTest plots of observed log2 fold differences across different Mandard grades of treatment response. FDR < 0.05 are indicated by the vertical dotted lines, red arrows highlight cell types of interest. Sample sizes as indicated below each plot.

To determine the cellular microenvironment of EAC we performed single cell RNA sequencing (scRNA-seq) on normal (*n* = 16) and tumour samples (*n* = 28) obtained from 26 patients, consisting of 13 samples from treatment-naïve patients, 6 from patients treated with neoadjuvant chemoradiotherapy (CRT) and 9 from patients treated with neoadjuvant chemotherapy (Table S1/S2 and Fig. S1A). These samples were representative of contemporary cohorts and reflect the clinical profile of EAC patients treated at University Hospital Southampton. We clustered single-cell transcriptomes from normal oesophagus and treatment-naïve cancer samples and cells formed a diverse set of communities that represented the major cell lineages present in the oesophagus. This data was used as a reference dataset to integrate (using canonical correlation analysis) and map single-cell transcriptomes from the chemo- and CRT-treated samples with a prediction score of >0.7 from label transfer. This ensured that cluster identification was robust and limited confounding by treatment or experimental confounding. We retained 32,677 cells (Fig. 1a) for analysis and classified cells into 10 cell lineages using canonical marker expression (Supplementary Data 1). Cancer cells, fibroblasts and T lymphocytes were the most abundant cell types recovered. We observed that fibroblasts consisted of both normal and cancer-associated cell sub-clusters that changed following treatment (Fig. 1b, see black arrows). Canonical marker expression confirmed this cell cluster as a Decorin (*DCN*) expressing cell population that was distinct from pericytes expressing *RGS5* (Fig. 1c). In comparison to normal oesophagus-derived cells, there were significant increases in macrophages, plasma cells, B cells and T-cells in untreated tumours suggesting a substantial immune infiltrate in EAC in general (Fig. 1d). As TRG3 is not associated with a clinically meaningful local response to NAC (neoadjuvant chemotherapy) [13], we chose to exclude data from TRG3 cases in our downstream analyses of treatment response. Comparing untreated tumours to treated tumours stratified by Mandard tumour regression grade (TRG), we observed consistent changes in fibroblast and plasma cell proportions in patients with better responses (TRG1/2 vs. 4/5) to either neoadjuvant CRT or chemotherapy (Fig. 1e).

Our previous work had identified fibroblasts, and CAFs in particular, as being tumour-promoting and chemoprotective in EAC [7]. Therefore, we questioned whether CAF phenotypes were heterogeneous in patients with different responses to neoadjuvant therapy and whether neoadjuvant chemo or CRT may damage or alter the function and phenotype of these clinically relevant stromal populations. To do this we analysed CAFs separately to better characterise the gene expression changes following neoadjuvant therapy.

### Treatment alters CAF gene expression profiles

As overall fibroblast proportions were significantly altered following a clinical response to neoadjuvant therapy, we performed differential expression analysis using MAST [14] stratified by treatment status and/ or type (Chemo/CRT) for the CAFs identified in Fig. 1b to assess for potential drivers of phenotypic changes in CAFs (Supplementary Data 2). A recent consensus map of human tumours identified cancer-associated fibroblast expression metaprograms (MPs) conserved over tumour types [15], for parity we refer to these metaprograms in our over-representation analyses (ORA) described below.

The proportions of CAFs were altered (above FDR) following treatment (chemo or CRT) in comparison to untreated tumour samples (Fig. S2A). Using an adjusted *p*-value (Benjamini-Hochberg) of 0.05 and a log2-fold change cut off set to 0.25, we identified 39 genes significantly upregulated in untreated CAFs and 52 genes significantly upregulated in treated CAFs (Fig. S2B). In untreated CAFs, over-representation analysis identified enrichment for genes involved in the AP-1 and fibrinolysis pathways and significant overlap with a published co-expression-derived cell type signature for mesenchymal stromal cells [16]. Regarding the consensus CAF MPs, the CAF10 and hypoxia programs defined the untreated CAFs (adjusted *p* values 8.7 × 10^−14^ and 5.2 × 10^−8^, respectively. Supplementary Data 3). The top five upregulated genes (*MMP1, COL10A1, CXCL8, TM4SF1* and *PLAU*) have been associated with cancer progression and metastasis-inducing mechanisms [17–20]. Following treatment, over-expressed genes were enriched for extracellular matrix organisation and wound healing pathways and overlapped a published co-expression derived cell type signature for adventitial fibroblast cells, suggesting a change in CAF states after treatment [21]. Upregulated genes in the treated cells were assigned to the published CAF1 and complement MPs (adjusted *p* values 4.5 × 10^−14^ and 2.1 × 10^−51^, respectively. Supplementary Data 3). The top five upregulated genes (*C1QTNF3, ANGPTL1, OGN, COL14A1* and *CXCL*12) have been previously associated with predictive signatures for chemotherapy resistance [22–26]. In the chemotherapy versus untreated CAF comparison, we identified 107 genes significantly upregulated in untreated CAFs and 71 genes significantly upregulated in Chemo treated CAFs (Fig. S2C). In the CRT versus untreated CAF comparison, we identified 41 genes significantly upregulated in untreated CAFs and 111 genes significantly upregulated in CRT treated CAFs (Fig. S2D). Thirty-eight genes were upregulated in both the Chemo and CRT treated CAFs with a top hit for the CAF complement MP in ORA (adjusted p value 5.3 × 10^−37^. Supplementary Data 3). Interestingly, the master regulator of CAF state, transcriptional factor 21 (*TCF21*) was significantly over expressed in both CRT and Chemo treated CAFs, suggesting re-programming of the CAF phenotypes following exposure to neoadjuvant therapy in vivo [27]. The differences between Chemo treated versus CRT treated CAFs were more striking, with 78 versus 235 over-expressed genes, respectively (Fig. S2E). Here, over representation analysis highlighted differential activity of the fibroblast stress and complement MPs in CRT versus Chemo-treated CAFs (adjusted *p* values 3.5 × 10^−19^ and 2.1 × 10^−13^, respectively. Supplementary Data 3). Fibroblast complement activation induced by chemotherapy has been associated with immunosuppression and metastatic relapse in Breast Cancer [28]. Interestingly, within the top five most over expressed genes in either treatment arm there were genes functionally important for radio-resistance or chemo-resistance and immunosuppression, namely *HILPDA* and *GSN*, respectively [29, 30]. Together, this data highlights the diverse effects of neoadjuvant therapy on fibroblast cells. Therefore, we hypothesised that fully characterising the diversity of fibroblast phenotypes would enable us to understand their roles in the dysregulation of the tumour microenvironment following systemic therapy with either Chemo or CRT.

### CAF phenotypes associated with treatment response

**Fig. 2:**
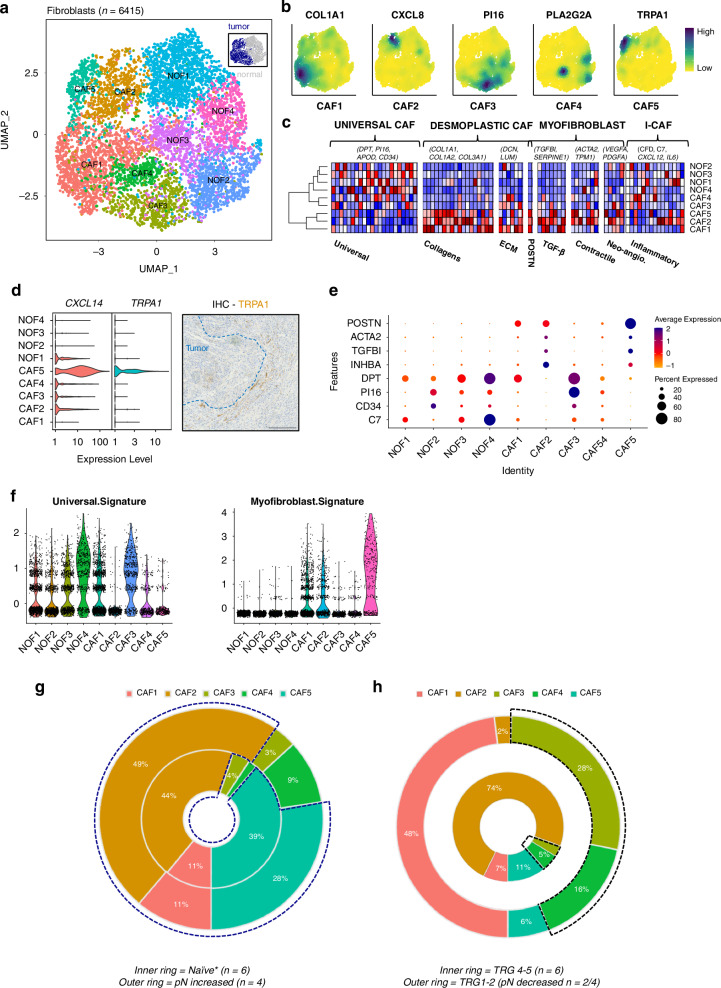
Cancer-associated fibroblast phenotypes in EAC. **a** UMAP of fibroblast populations, inset UMAP identifies sample origin. **b** UMAPs of the CAF populations with selected lineage markers. **c** Heatmaps of canonical marker gene expression and selected cluster markers displaying universal, desmoplastic, myofibroblast and inflammatory CAF phenotypes. **d** Violin plot of *CXCL14* and *TRPA1* RNA expression and a representative IHC image of TRPA1 staining at the tumour/stroma boundary. **e** Dot plot of marker gene expression for fibroblast clusters. **f** Violin plots of the universal fibroblast signature and the myofibroblast signature across the NOF and CAF clusters. Fibroblast changes following treatment. **g** Neoadjuvant treatment naïve patients. The outer ring are the four cases with an increased in their pathological node status following surgery. The blue dashed line surrounds the myoCAF populations. **h** Chemo/CRT treated patients, stratified by different TRG statuses as indicated. The black dashed line surrounds the CAF3 and four populations.

We identified 6415 fibroblasts (*DCN+* and/or *VIM+* cells), clustering into four normal-associated fibroblast (NOFs) and five cancer-associated fibroblast groups (CAFs) based on their variable expressed genes (Fig. 2a; Supplementary Data 4). NOF1-4 clusters were marked by expression of *PTGDS/APOD* (NOF1)*, PCOLCE2/SLPI* (NOF2), *MT1X/MT2A* (NOF3) and *C7/APOD* (NOF4) and CAF1-5 clusters by expression of *COL1A1/COL3A1* (CAF1), *IGFBP3/CXCL8* (CAF2), *SFRP4*/*PI16* (CAF3), *PLA2G2A/SFRP2* (CAF4) and *TRPA1/F3* (CAF5), respectively (CAF markers shown in Fig. 2b). Focusing on the CAF clusters, CAF1, 2, and 5 expressed *ACTA2* (α-SMA) and *POSTN* in comparison to CAF3 and 4 and the NOF clusters (Fig. 2c/e). These three *POSTN*+ clusters represent myofibroblastic CAF (myoCAF) phenotypes that are prognostic and can promote tumour invasion in EAC [7]. CAF1 markers, *COL1A1* and *COL3A1* encode components of the extracellular matrix with an important role in adhesion and differentiation [31]; whereas CAF2 expressed *IGFBP3* and *IL8* that are known to regulate IGF signalling and angiogenesis [19]. CAF5 expressed *F3* (CD142/tissue factor), *CXCL14* and Transient Receptor Potential Cation Channel Subfamily A Member 1 (*TRPA1)*. TRPA1 expression was observed in CAF5 cells from either treated or naïve samples and IHC confirmed expression in stromal regions (likely to be CAFs) adjacent to tumour cells (Fig. 2d). Interestingly, CD142 is normally only expressed in subendothelial cells and *TRPA1*, also known as the Wasabi receptor, is a Ca^2+^ dependent membrane bound stress sensor [32]. Interestingly, restricted *TRPA1* expression was observed in the Podoplanin (*PDPN*+) selected and adipogenic (*PTGDS*+) EAC-derived CAF phenotype (our CAF5) recently described by Croft et al. [10] and we also observed their COL1A2+ (CAF1) and MFAP4+ (CAF3) populations (Fig. S3), suggesting that CAF phenotypes may be conserved after NAT across EAC cohorts. CAF3 shared many expressed genes with the inflammatory CAFs (iCAFs) identified in pancreatic ductal adenocarcinoma (PDAC) (Fig. 2c) [33, 34]. CAF3 also expressed *PI16* and *DCN*, genes associated with a detox-iCAF population identified by Kieffer et al. [35] while the presence of Decorin (*DCN)* suggests that CAF3 may be related to desmoplastic CAFs, a phenotype that can overlap with iCAFs [36]. CAF4 shared its top marker gene *PLA2G2A* (phospholipase A2 group IIA) with metabolic CAFs previously identified in PDAC [37]. Furthermore, they clustered with NOFs and next to CAF3 when considering CAF-associated gene-sets curated by Qian et al. [38] (Fig. 2c). Focusing on the NOF clusters, NOF2 and 4 expressed *PI16, DPT*, and *CD34* (Fig. 2E). Fibroblasts with these characteristics have been described as universal fibroblasts [39]. Interestingly, CAF3 shared many similarities with NOF2 and 4 (Fig. 2c) and had a high universal fibroblast score concomitant with the lowest myofibroblastic score (Fig. 2f).

In neoadjuvant treatment naïve tumours CAFs 1, 2, and 5 (all myoCAF) were the dominant populations recovered (Fig. 2g). Overall, CAF1, 2 and 5 cells were detected in 24/26 patients and after excluding samples with low total CAF counts (*n* < 50), CAF1, 2 and 5 cells were on average 76% (95% CI: 55–96) of all CAF subtypes. CAF2 and 5 on their own equated to ~36% (95% CI: 10–62) of all CAFs present (Supplementary Data 5), suggesting myoCAF are ubiquitous and present in most tumours before surgery. To establish a deeper understanding of the complexity of CAF dynamics during NAT we re-analysed and expanded the data presented in Fig. 1a, b to focus on CAF heterogeneity during phenotypic transition in response to NAT (Fig. S4A-C). In chemotherapy treated tumours the TME was dominated by CAF1 and CAF3, whereas after CRT mixed populations of CAF were recovered with one patient’s tumour (a non-responder, TRG4) dominated by CAF2 (Fig. S4D). This suggested that CAF dynamics were not only determined by NAT treatment type, but also by response to treatment. Therefore next, we explored how CAF heterogeneity was determined by response to treatment. Following either neoadjuvant chemo/CRT and surgery, non-responding tumours (TRG4-5 cases) demonstrated more CAF2 cells (74 vs. 44%) and retained a significant fraction of CAF5 cells (11%). By comparison, in responders (TRG1-2) CAFs 1, 3 and 4 made up most of the CAF cells recovered (Fig. 2h). The CAF1 population made up approximately half of all the CAFs recovered from responders. The CAF1 cluster contained cells from 21 patients, where 96% were from treated samples and enriched in two patients, both responders to treatment. This suggests that CAF1 persists and/or expands in response to neoadjuvant therapy, regardless of treatment type, and after tumour regression. The CAF3 cluster was derived from 14 cases, where ~90% of the cells were obtained from tumour samples following chemotherapy. The CAF4 population, recovered from 15 patients, was predominantly enriched in samples from a subset of cases treated with CRT (82% of all CAF4 cells from 4 patients), suggesting CAF heterogeneity may be further associated with neoadjuvant treatment type (Supplementary Data).

**Fig. 3:**
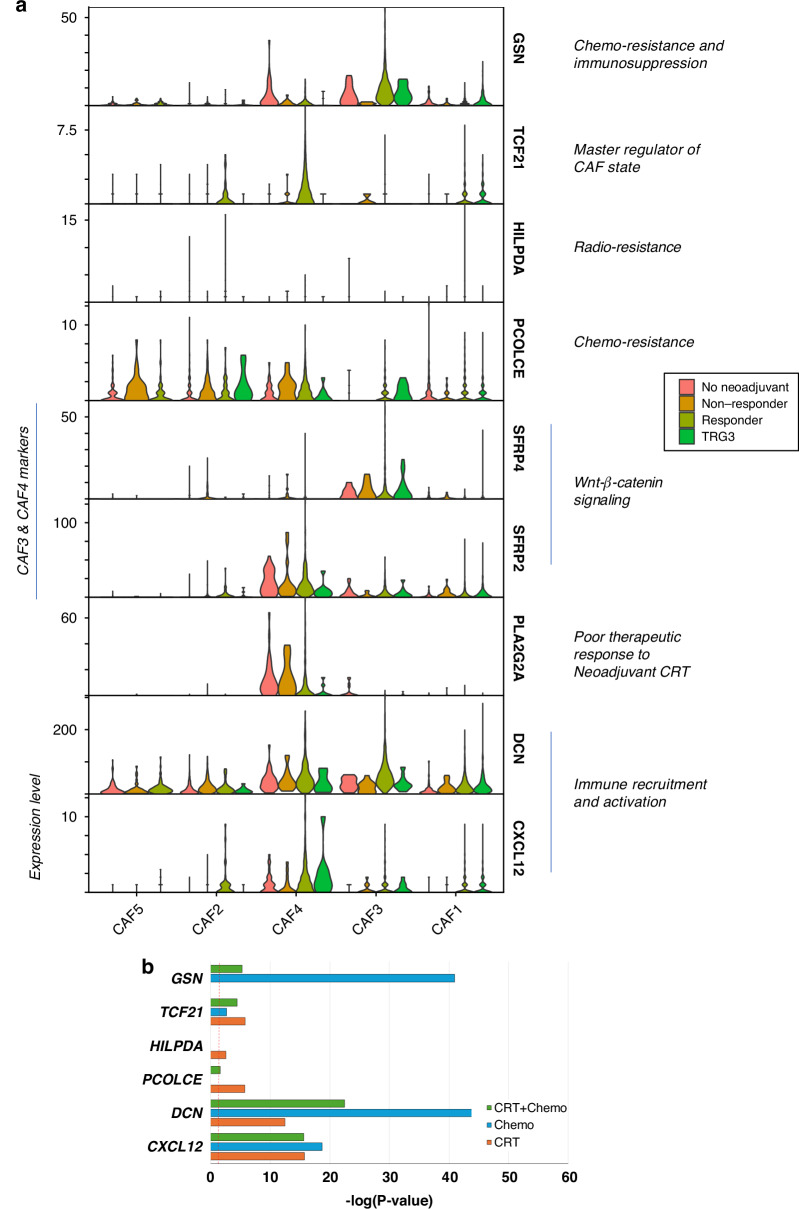
Candidate genes associated with neoadjuvant response in EAC CAFs. **a** Violin plots of gene expression in individual CAF cells. The five CAF clusters are labelled accordingly and stratified by treatment response. **b** Differential expression of candidate genes (from panel A, excluding CAF3 & 4 marker genes) comparing CAFs exposed to neoadjuvant treatment (as indicated in the chart legend) versus CAFs from surgically resected tumours without neoadjuvant therapy. The bars represent −log10(*p*-values) and the red dotted line is the *p*-value cut-off at 0.05.

Having established significant heterogeneity in CAF sub-type populations determined by neoadjuvant treatment type, we next explored the potential transcriptomic drivers of this phenomenon. A good response (TRG 1-2) to neoadjuvant therapy was associated with broader expression of *TCF21* and Gelsolin (*GSN*) transcripts in CAF clusters 1, 3, and 4 (Fig. 3a). Conversely, Procollagen C-endopeptidase enhancer (*PCOLCE*) expression levels were higher in the CAF2 and 5 clusters from both untreated and non-responding patient samples; this gene is a marker for CAF infiltration, chemo-resistance and poor outcome in a previous pan-cancer study [40]. Secreted frizzled-related protein 4 (*SFRP4*) and 2 (*SFRP2*) were the top marker genes for the CAF3 and 4 populations that were enriched in samples from responders to neoadjuvant therapy. A previous study has shown that restoration of SFRP4 secretion by pancreatic stellate cells reduced Wnt–β-catenin signalling in cancer cells and their invasive ability [41]. *DCN* and *CXCL12* were overexpressed by our chemo/CRT remodelled CAFs (CAFs 3 and 4) (Fig. 3b), CAF populations like these were found to be expanded in NAC (neo-adjuvant chemotherapy) responsive rectal cancers and are known to activate anti-cancer immunity and suppress tumour progression [42]. These treatment remodelled CAFs shared the universal fibroblast markers (*DPT*, *PI16* and *CD34;* See Fig. 2c/e) that were seen in the rectal CAF populations, suggesting that these may be functionally similar cell states with immunomodulatory potential. These data suggest that different CAF states are associated with neoadjuvant treatment response, and we identified a TRPA1 expressing population of myoCAFs that persist following NAT.

### Inferred cellular communication associated with non-response to neoadjuvant therapy

**Fig. 4:**
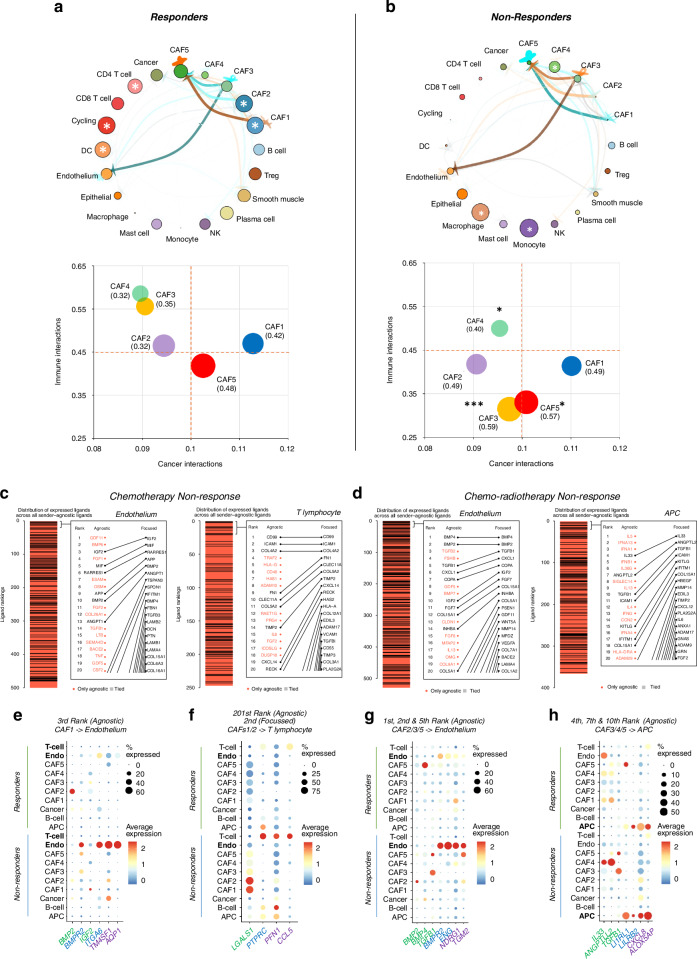
Cell communication analysis of OAC tumours following neoadjuvant therapy. **a** Top panel—Comparative Cell-Cell Interaction network in responders (*P*-value filter set at 0.2), where node size (Page rank odds-ratio) and edge thickness (% of interactions) represents importance of the cell communication between cells. The arrows indicate the signal direction and colour the activation status (Brown = Up and Blue = Down). The white asterisks indicate the top ranked cell types for NAT response in either patient group. Bottom panel - Bubble plots of cancer associated fibroblast interaction network changes (expressed as proportions) of cancer (x-axis), immune (y-axis) and stromal compartments (bubble radius) associated with NAT response. The asterisks indicate statistically significant differences in proportions between response groups (Two by Two Chi-squared test, two tailed Fisher’s Exact *P*-values; * ≤0.05, ***≤0.001). **b** As (**a**) for non-responders. Line plot of the distribution of ranked ligands for the indicated receiver cell type in the sender agonistic (all cell lineages) and sender-focused (CAF1-5) NicheNet analysis in non-responders to chemotherapy (**c**) and chemo-radiotherapy (**d**). Ligands in red are not in the top ranked ligands for all CAF-senders. APC antigen presentation cell. **e**, **f** A scaled DotPlot of target gene candidates across cell types stratified by chemotherapy response type. Colour scale of average expression and black circles denote the percentage of expressing cells. **g**, **h** A scaled DotPlot of the target gene candidates across cell types stratified by chemo-radiotherapy response type. Colour scale of average expression and black circles denote the percentage of expressing cells. Gene symbol colours: Green = Ligand; Blue = Receptor; Purple = target gene.

We now questioned how the EAC tumour microenvironment signalling network was modulated following therapy. To address this, we reconstructed ligand-receptor (LR) interactions by CellPhoneDB and utilised network-based analyses (CrossTalkeR) to prioritise changes in the cancer, immune and stroma compartments associated with neoadjuvant therapy response [43, 44]. Inferred cell-cell interaction networks were constructed for both the responder (TRG 1 + 2) and non-responder (TRG 4 + 5) patient groups and compared to untreated tumour samples (Supplementary Data 6). The top five important cell populations in responders were dendritic cells, CD4 T cells, cycling cells (expressing T cell markers) and CAFs 1 and 2 (Fig. 4a—top panel). Conversely, in non-responders, monocytes, macrophages and CAF4 were highlighted as important contributors (based on random-walks estimates with page rank odds-ratios) for the cellular network (Fig. 4b—top panel). CrossTalkeR analysis uses network topology measures to find cell populations sending signals (influencers), nodes receiving signals (listener) or both (mediators). In tumours from responders CAFs 1, 2 and 5 were listeners and CAFs 3 and 4 mediators. In non-responders, this topology was flipped and was associated with significant changes in the immune interaction partners for CAFs 3, 4 and 5 (Fig. 4a/b—bottom panels). This was further highlighted by differential ligand–receptor interactions associated with the response type. For example, there were changes in the predicted collagen-integrin signalling that dominated inter-CAF crosstalk (Fig. 4a—top panel); mutually exclusive activation of PDGF, VEGF or NOTCH signalling to endothelium and cytokine signalling via CXCL9, CCL11, CD70 and CCL18 to the immune system (Fig. S5A). The inferred cell-gene interaction network analysis identified several divergent interactions previously associated with chemoresistance, where signalling via Amphiregulin (*AREG*), Fibronectin (*FN1*), CD52 and CD55 from myeloid cells, CAFs, leucocytes and cancer cells respectively, was increased in non-responders (Fig. S5B) [45–48].

So far, our findings indicated that the activity of individual CAF populations was both complex and context specific and dependent on their immediate signalling network in the tumour microenvironment. To gain further insight, we used a NicheNet analysis to prioritise important CAF subtype interactions associated with non-response to chemotherapy or chemo-radiotherapy and CrossTalkeR to visualise changes to the network topology (Fig. S5C). The predicted cell-cell communication (CCC) effects of each treatment modality could highlight potential resistance mechanisms to the different neo-adjuvant treatments. To do so, we constructed sender-focused NicheNets for each CAF subtype and set receiver cell types as antigen presenting cells (APCs), B lymphocytes, Cancer, Endothelium and T lymphocytes, respectively. Top ranked CAF-specific ligands were then assessed in a mirrored set of sender-agnostic NicheNets to determine the likelihood that the ligand is important in the global CCC process. Finally, causality was inferred by investigating alterations to predicted target gene expression in the receiver cell types.

In non-responders to neoadjuvant therapy, CAF to endothelium signalling was highly ranked in both the sender-agnostic and CAF subtype-focused NicheNets (Fig. 4c, d). In chemotherapy non-responders, CAF1 signalling via a predicted *IGF2-ITGA6* interaction correlated with upregulation of target gene expression (*TM4SF1* and *AQP1*) in endothelial cells following chemotherapy (Fig. 4e). Clinically, elevated IGF2 levels in TNBC tumours has been shown to correlate with adverse prognosis and resistance to anti-PD1 immunotherapy [49]. TM4SF1 has been shown to mark a subpopulation of endothelial cells with progenitor potential and highly angiogenic in nature [50].

In CRT non-responders, CAF2 signalling through *BMP2-BMPR*2 and *TGFB1* (through various receptors) correlated with upregulation of several target genes (i.e., *NDRG1, TGM2* and others) in endothelial cells (Fig. 4f). BMP2 is a member of the TGF-β superfamily of growth factors, that is known to prevent apoptosis via the BMP receptor 2 (BMPR2) in embryonic fibroblasts and BMPR2 has been shown to act as a gatekeeper to protect endothelial cells from activating TGFβ responses [51, 52]. The target gene expression of *TGM2* and NDRG1 has been linked to endothelial inflammation, remodelling and angiogenesis [53, 54].

Remaining with CRT non-responders, we observed highly ranked CAF4 (enriched in CRT treated cases) and CAF5 interactions with antigen presenting cells (APCs) via *IL33-IL1RL1, ANGPTL2-LILRB2* and *TGFB1-ITGB1* was associated with changes in downstream target gene expression (Fig. 4g). Correspondingly, interleukin-8 (*CXCL8*) and *ALOX5AP* was overexpressed in APCs from non-responders. It is well-established that tumour associated macrophages are the main source of interleukin-8 in the tumour microenvironment and in EAC it appears that specific CAF subtypes may be skewing macrophage polarisation in CRT non-response [55].

Returning to the chemotherapy non-responders, prioritised immune cell interactions with CAF subtypes included T lymphocytes (2nd ranked across all receiver cell types), APCs and B lymphocytes cells in that order in the CAF-focused NicheNets. This high ranking was not maintained in the sender agnostic NicheNet (201st ranked), suggesting other cell-type interactions are more important. In keeping with this, we observed up-regulation of *PFN1* (Profilin1) and *CCL5* in T lymphocytes from non-responders, suggesting cytotoxic dysfunction in a proportion of the T lymphocytes [56, 57] (Fig. 4h). The 2nd ranked ligand-receptor interaction from the CAF-focused NicheNets involved *LGASL1-PTPRC* in the CAF1 and 2 subtypes, suggesting some subtypes are inducing T cell death in non-responders [58] (Fig. 4h).

### Stromal cell populations with altered TRPA1 and gelsolin expression surround tumour nests

**Fig. 5:**
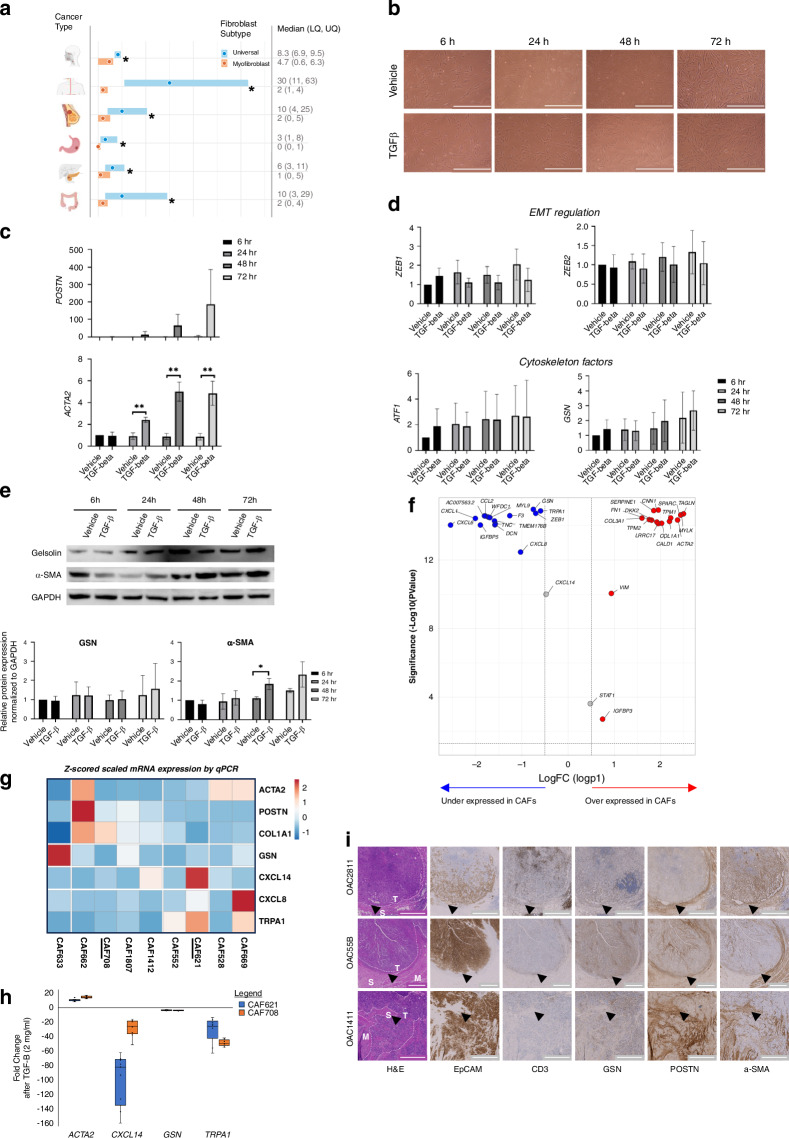
In vitro validation of gelsolin expression and myofibroblast programs in ex vivo patient-derived CAFs and EAC tumour tissue sections. **a** *GSN* expression in the universal and myofibroblast populations across head and neck, oesophageal squamous, breast, gastric, pancreatic and colorectal cancer (*p < 0.0001). **b** HFFF2 fibroblasts treated with TGF-β for 72 h show CAF differentiation compared to vehicle (**c**) Relative expression of mRNA of myofibroblast genes *ACTA2 and POSTN* following CAF differentiation by TaqMan assay at 6 h, 24 h, 48 h and 72 h compared to vehicle (*n* = 3 replicate experiments). **d** Relative expression of mRNA of *ZEB1 and ZEB2* (EMT regulation)*, ATF1* and *GSN* (cytoskeleton factors) following CAF differentiation by TaqMan assay at 6 h, 24 h, 48 h, and 72 h compared to vehicle (*n* = 3 replicate experiments). **e** Protein expression of GSN, αSMA and GAPDH a in TGF-β treated HFFF2 cells and vehicle quantified by Western Blotting and densitometry in biological triplicates (unpaired two-tailed t-test). **f** Volcano Plot of top ten over and under expressed genes and selected differentially expressed GOIs in patient-derived ex-vivo CAFs co-cultured with EAC cancer cell line, MFD1. Data from ref. [4]. *GOIs/Genes of interest include GSN; myoCAF markers: POSTN, ACTA2; EMT marker: ZEB1, VIM; CAF1 markers: COL1A1, COL3A1; CAF2 markers: CXCL8, IGFBP3; CAF5 markers: CXCL14, TRPA1, F3; Predicted transcription factor regulators: MYLK, STAT1. Adjusted *P* value cutoff set to an alpha of 0.05 (1.3 in –log10 space) and LogFC cutoff set to ±0.5. **g** Clustered heatmap of patient derived CAF mRNA expression heterogeneity by qPCR. Z-scored expression values as indicated by the colour scale. Underlined CAF samples were used for experiments shown in (**c**). **h** Fold change in mRNA expression in selected patient derived CAFs at 72 h following TGF-beta treatment. Median line shown on boxes. **i** Representative low power images of IHC expression patterns in EAC in the same tissue area for GSN, POSTN, α-SMA, CD3 and EpCAM from adjacent sections, and the corresponding H&E stain. H&E images are annotated with white dashed lines to label major tumour regions (T), stroma (S), and smooth muscle (M), where present. Three representative patients are shown. Example peritumoral fibroblast areas are marked with arrowheads. Scale bars—1000 μm.

We sought to characterise the expression of several established CAF markers and these candidate proteins, in patient-derived CAFs and tumour tissues. Our single cell data and evidence from other cancers (Fig. 5a) highlighted a relationship between fibroblast subtypes (myofibroblast versus universal fibroblasts) and altered expression of *GSN* (See Fig. 3) and therefore, may be of particular importance for response to neoadjuvant chemotherapy. Alongside the established EAC myoCAF markers alpha-SMA and POSTN, we chose to examine GSN and TRPA1. Geloslin, a known tumour suppressor gene, is a multifunctional regulator of cell structure and GSN downregulation in a glycoprotein panel is a diagnostic biomarker for Barrett’s oesophagus requiring clinical intervention [59]. TRPA1 was chosen because its expression was exclusive to the myoCAF sub-population (CAF5) that we observed to be present in EAC before therapy and to persist in non-responders (Fig. 2d). Additionally, TRPA1 has the potential to be targeted as it is a cell surface molecule known to regulate cellular calcium influx and it can control fibrosis via fibroblast activation [60]. Initially, we investigated *GSN* in human fibroblast (HFFF2) cells following TGF-β exposure. TGF-β treated HFFF2 fibroblasts displayed visually had a myofibroblastic phenotype (Fig. 5b). mRNA and protein expression of *ACTA2, POSTN;* our candidate gene *GSN* and its known regulator, *ATF1* [61] were measured from 6 to 72 h. Accordingly, *POSTN, ACTA2* (α-SMA) increased and plateaued at 6 h and 24 h, respectively (Fig. 5c); however TGF-β did not induce downregulation of *GSN* or modulate *ATF1* levels (Fig. 5d). Protein expression of α-SMA increased at 48 h, but there was no change in GSN (Fig. 5e). Re-analysis of our previously published scRNA-seq data [8] of ex vivo patient-derived CAFs co-cultured with MFD1 EAC cells, demonstrated lower expression of *GSN* and *TRPA1* and overexpression of *ACTA2* and *MYLK*, a predicted regulator of *POSTN* and *ACTA2* expression, that is activated by the Ca2 + - calmodulin complex (Fig. 5f). In patient-derived CAFs, TGF-β treatment induced *ACTA2* and reduced both *GSN* and *TRPA1* mRNA expression (Fig. 5g/h). In EAC tissue, TRPA1 expression was localised to subpopulations of CAFs surrounding tumours, presumed to be CAF5 (Fig. 2d), whereas *GSN* was more broadly expressed, localising to both intratumoral fibroblast and lymphocyte populations (Fig. S6). Tumour epithelium heterogeneously and weakly stained in a few cases, but most tumours did not express GSN (74%, *n* = 14/19), in keeping with its role as a tumour suppressor. We compared GSN expression to adjacent sections of tumour stained for POSTN, α-SMA, CD3 and EpCAM (Fig. 5i). Surrounding tumour nests, we found distinct peritumoral stromal regions positive for POSTN/α-SMA (i.e., myoCAFs) that did not appear to express GSN, and sub-populations expressing TRPA1. Together, this confirmed the presence of specific myoCAF populations in distinct cellular neighbourhoods in tumours in situ, the phenotype of which is not fully recapitulated in vitro.

### A cancer-associated myofibroblast—fibroblast signature is prognostic across cancers

**Fig. 6:**
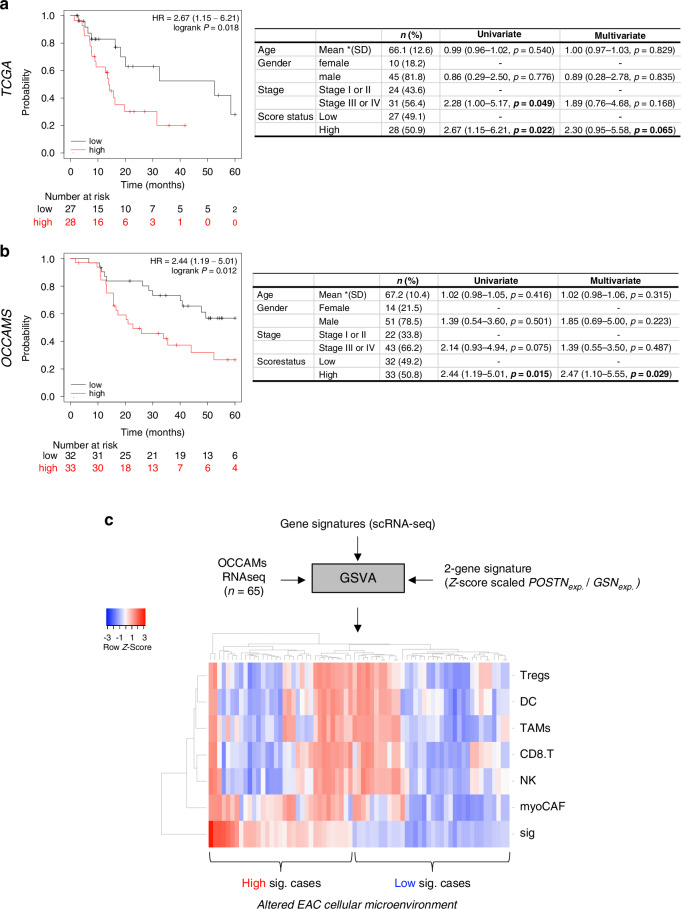
Prognostic value of the 2-gene expression model in EAC genomic consortium cohorts. Kaplan-Meier survival curves (Black line: 1st tertile scores (“Low group”). Red line: 3rd tertile scores (“High group”) and Cox Proportional Hazards tables for our 2-gene expression signature in the OAC cases from The Cancer Genome Atlas project (**a**) and OCCAMs consortium (**b**). Overall survival follow-up to 60 months from diagnosis. Only cases with complete data for all covariates were included in the multivariate analysis (**c**) Heatmap of the scRNA-seq derived cell signature scores from GSVA analysis of OCCAMs cases with whole genome expression data. Euclidean distance and average linkage were used to clustered samples. The curly brackets indicate cases with either high or low 2-gene signature scores from (**a**).

To elucidate the clinical potential of this new biological knowledge, we validated the risk prediction performance of a 2-gene (*GSN* and *POSTN*) expression score calculated from the z-score scaled ratio of *POSTN* to *GSN* expression (myoCAF versus non-myoCAF) for overall survival in EAC. In TCGA EAC cases, a high expression score (3rd tertile) predicted a significantly shorter OS with a median survival time of 13.9 months versus 52.5 months in comparison to lower expression scores (HR = 2.67, 95% CI: 1.15–6.21, *P* = 0.018) (Fig. 6a). When considering the other pan-cancer datasets from the TGCA study, our expression signature was statistically significant (*P* ≤ 0.05) for OS in 6 out of 21 cancers and associated with a poorer outcome (Table S3).

We then applied our 2-gene expression score to OCCAMs consortium cases analysed by bulk tumour RNA sequencing (*n* = 139 cases) [62]. In this cohort, cases with a high or low expression score (HR = 2.44, 95% CI: 1.19–5.01, *p* = 0.012) achieved a median OS of 15.7 and 30.0 months, respectively (Fig. 6b). By multivariate analysis, including additional covariates, such as age, gender and pathological staging, the 2-gene signature retained prognostic independence in the OCCAMs cohort (HR = 2.47; *p* = 0.029) and the smaller TCGA cohort trended towards significance (HR = 2.30; *p* = 0.065). We used our scRNA-seq-derived cell signatures in a Gene Set Variation Analysis (GSVA) to deconvolute the bulk RNA-seq data into myoCAF, CD8 + T cell, Treg, NK, M2-like macrophage (TAMs) and DC associated gene sets and clustered OCCAMs cases by their expression score. Patients with high scores (associated with shorter OS) clustered into distinct groups with clear enrichment for myoCAF and immune cell signatures, suggesting this prognostic score can robustly identify cases with a myoCAF enriched cellular landscape (Fig. 6c).

## Discussion

We demonstrate for the first time that treatment influences CAF phenotype and TME composition in EAC and identified a myoCAF (CAF5) that persists in non-responders to NAT. We show that specific CAF phenotypes are associated with treatment response and that these may differentially influence cellular signalling networks. Finally, we show that a 2-gene fibrotic gene signature can be used to prognosticate in EAC cohorts and may be informative across several pan-cancer datasets. Much is now known about the genotypic and phenotypic changes in EAC cells after neoadjuvant treatment (NAT). Despite this, we have been unable to fully explain differential responses between patients and between treatment modalities (chemo versus CRT) and little is known about the concomitant changes in the TME. Perhaps more disturbing is the realisation that a complete pathological response to neoadjuvant CRT does not confer the same survival advantage as a complete pathological response after chemotherapy [63]. This clinical data points to other elements in the EAC ecosystem as determinants of outcome after neoadjuvant treatment. We set out to explore this, with a primary focus on the most abundant TME cell type, cancer-associated fibroblasts. We have made several findings that may begin to explain some of the clinical complexity of EAC.

Firstly, we observed changes in the constitution of the EAC TME in response to NAT and key differences with the type of NAT received. Whilst this is unsurprising, we also documented specific changes in fibroblast biology related to treatment response and identified specific CAF gene expression changes associated with NAT type. Three of our CAF phenotypes were consistent with a recent single cell transcriptional study of EAC in 8 patients, four of which received NAT with four cycles of FLOT [10]. Consistent with the prior study, we observed an increase in complement expressing CAFs (CAF3) associated with chemotherapy treated tumours. Given our larger sample size, we observed an additional angiogenesis-associated (*CXCL8*+) myofibroblast phenotype (CAF2) and a CAF4 (*PLA2G2A*+) population associated with both chemoradiotherapy and a good pathological response at follow up. Interestingly in pancreatic cancer, *PLA2G2A* + CAFs were found to attenuate the antitumor ability of tumour infiltrating CD8 + T cells [64]. At the individual patient level, our data suggests that the type of NAT prior to checkpoint inhibition may reduce its future efficacy because of these selective changes in the tumour stroma.

By interrogating EAC CAF biology we identified five CAF clusters, including three myoCAF phenotypes (CAF 1, 2, and 5). These myoCAFs dominate the untreated EAC TME and CAF1 persists after treatment in tumours that respond to NAT. CAF1 has features of the COL1A1-expressing CAF type known to restrict tumour growth [31] and is most likely related to wound healing in this context. Conversely, CAF2 and CAF5 dominate the TME of non-responders and we were able to observe GSN negative and TRPA1 expressing stromal cells adjacent to EAC cancer cells in human specimens. They appear central to stromal crosstalk with cancer cells and the endothelium. In our cohort, poor response to NAT was associated with CAF-derived IGF2 or BMP2 signalling to endothelial cells and diverging immunosuppressive signals directly to lymphocyte and myeloid cells, resulting in detectable changes in their gene expression following surgery.

What determines the persistence of one myoCAF subtype or another after NAT, and whether this is driven by cancer cells or the stroma, or another factor, is yet to be determined, but we have gained some insight from human tumour analysis. From the data we can speculate the following: 1) All myoCAF subtypes were positive for *POSTN* expression, suggesting a shared origin [65] and; 2) these populations may represent the cycling of different transitional cell states from early-activated tumour-proximal myoCAFs (CAF5), to more terminal differentiated myoCAFs (CAF2) to a population of less contractile and more ECM/ pro-collagen producing cells (CAF1); 3) or these cell phenotypes may represent myoCAFs residing in specific tissue niches.

The proximity of CAF5 to cancer cells and the endothelium makes this myoCAF subtype of particular interest. CAF5 are exclusive in their TRPA1 expression, a molecule known to regulate calcium influx and can control fibrosis via fibroblast activation [66]. Intriguingly, a recent study of idiopathic pulmonary fibrosis has demonstrated a role for TRPA1 in both fibrotic modulation and M2 macrophage polarisation control [60]. We can speculate that targeting TRPA1 has translational potential, as inhibition could potentiate both the stromal (myofibroblast) and suppressive immune (myeloid) compartments identified to be important nodes in the cell communication topology of non-responders in this study. A study in prostate cancer CAFs identified that TRPA1 activation by resveratrol leads to strong Ca^2+^ cytosolic influx and secretion of VEGF and HGF [67]. In the same study, activation of TRPA1 in a co-culture model reduced prostate cancer cell death by 40%. In our study, EAC CAFs also expressed *VEGF*, *VEGFR* and *HGF* along with other growth factors that had some importance in our interaction network (data not shown), suggesting that TRPA1 may function similarly in EAC. Contrastingly, AITC-induced TRPA1 activation augmented ERK1/2 phosphorylation in cultured lung fibroblasts, which in turn inhibited TGF-β receptor signalling [32]. Therefore, TRPA1 targeting agents should be employed to investigate the mechanism of CAF activation and myofibroblast differentiation further.

We identified that *GSN* gene expression, as part of a myofibroblast-fibroblast score has prognostic utility in EAC and several other cancer types. Within EAC tumours and patient-derived CAF cultures we observed down-regulation of *GSN* and conversely, *GSN* was upregulated in responders to NAT in the residual CAF subtypes (CAF1, 3 and 4). The actin cytoskeleton promotes myofibroblast function during fibrogenesis and in tumour stroma remodelling. Numerous actin binding proteins are linked to cancer, leading to abnormal cytoskeleton architecture, instability and metastasis (Reviewed in ref. [68]). *GSN* is one such protein. Reduction of *GSN* in epithelial cells induces EMT and increases motility and invasiveness [10, 11] and in *GSN* null mice, dermal fibroblasts have excessive actin stress fibres and have increased contractility in vitro [12]. This supports *GSN* being a regulator of the myoCAF phenotype in EAC, with down-regulation of *GSN* required for myoCAF differentiation.

Our ex vivo data suggests that intimate cancer/CAF interactions and specific tissue states are required for the full induction of these myoCAF phenotypes in vivo. Our findings suggest that Ca^2+^ signalling via TRP receptors in specific tissue niches may also be important in myoCAF differentiation in EAC [69]. This is intriguing as it is known that loss of mitochondrial Ca^2+^ uptake promotes myofibroblast differentiation by epigenetic reprogramming [70] and Ca^2+^ signalling is implicated in CAF-induced drug resistance in ovarian cancer, but the key players of calcium signalling in EAC are unclear [71].

Our study has several limitations, it was not possible to obtain matched pre- and post-treatment samples for the entire cohort, only half of the chemotherapy treated patients received FLOT and there were differences in tumour location of CRT treated cases (83% vs. 53–6% distal oesophagus) compared to the untreated/chemo-treated cases. Although our study was performed on a relatively large cohort of patients considering previous single cell work in EAC, the number of patients in each treatment group is low and therefore we cannot exclude selection bias. Future digital pathological and/ or spatial transcriptomic investigations are probably required to provide a higher-resolution measurement of EAC CAF heterogeneity present in tumours following genotoxic and immunotherapies.

In summary, this study provides a compendium of cell phenotypes in EAC across the current NAT treatment pathway that provides insights into CAF biology and cancer progression. myoCAFs represent an axis to repurpose agents to enhance current therapies and immunotherapy. Further work will be required to define how cancer and myoCAFs interact in specific tissue niches and how to generate faithful model systems for laboratory investigations.

## Supplementary information

Supplementary Material

Figure S1

Figure S2

Figure S3

Figure S4

Figure S5

Figure S6

Supplementary data

## Data Availability

The scRNA-seq datasets supporting the conclusions of this article are available in the NCBI GEO repository, Accession number: GSE173950. The source data for all quantification and statistical analyses are provided with this paper in the Supplementary Information/Source Data file. All other data that support the findings of this study are available from the corresponding authors upon reasonable request.

## Code availability

Will be made available to reviewers and editors upon request and will be made public and freely available (https://github.com/mjrz/script--share) at publication.
