# Supplementary material for: Cancer-associated fibroblasts are associated with neo-adjuvant treatment response in oesophageal adenocarcinoma
Source: Br J Cancer. 2025 Jul 10;133(5):633–47. doi: 10.1038/s41416-025-03080-8 (PMC12405553; doi:10.1038/s41416-025-03080-8)
Supplement: Supplementary file 1 — Supplementary Material [file 41416_2025_3080_MOESM1_ESM.pdf]

EXPERIMENTAL MODEL AND SUBJECT DETAILS

Human samples - scRNA-seq

Human tissue samples were obtained from patients through University Hospitals Southampton NHS trust after informed consent. This study was approved by The Proportionate Review Sub-Committee of the North East - Newcastle & North Tyneside 1 Research Ethics Committee. (REC No: 18/NE/0234). Twenty-six patients with esophageal adenocarcinoma undergoing treatment with curative intent were recruited to our study. Samples of their tumor were taken at either a staging investigation (n=4) or resection (n=22). Samples from staging investigation consisted of six to eight 2x2mm endoscopic biopsies and samples from resection consisted of a single 8mm punch biopsy. These samples were collected in the operating theatre by a surgeon and immediately transported to the laboratory in a tissue storage solution (Miltenyi Biotech). Tissue was promptly disaggregated to a single cell suspension and analyzed using DropSeq (Macosko *et al.*, 2015).

METHOD DETAILS

Tissue disaggregation- scRNA-seq

Samples were relocated to the laboratory and placed in a tissue culture hood, placed in a petri dish and washed with PBS-A and then minced with a scalpel to approximately 1mm chunks. Minced tissue was transferred to a 50ml Falcon tube with 5ml of DMEM complete, 100 µl of 150 U/ml Collagenase P, 100 µl of 10 mg/ml DNase and 5ml of Trypsin. The samples were placed in an incubator at 37° C with agitation for 60 minutes and disturbed at 15 minutes and 30 minutes with pipettes. Samples were then passed through a 70 µm strainer and 10 ml of DMEM without supplements added. Cells were then pelleted using centrifugation and media aspirated. Cells were resuspended in RBC lysis solution and incubated at 4°C for 10 minutes, 10 ml of DMEM was added and the suspension was passed through a 40 µm strainer before being pelleted and resuspended in 1 ml of cell suspension buffer. 10 µl of cells in suspension

and 10 μl of trypan blue was mixed together and cells were counted on a C-chip Fuchs-Rosenthal haemocytometer. A 150,000-cell aliquot was made up to 1.5 mls with cell suspension buffer and prepared for DropSeq. Tumor samples were taken through to sequencing regardless of whether the normal sample was successful. Normal samples were only run if a matched tumor sample was available.

DropSeq Single cell RNA sequencing

Single-cells (100 cells/μL) and barcoded mRNA-binding micro-particles (ChemGenes Corp, 100 beads/μL) were coupled in ~1 nL droplets containing cell lysis buffer (200 mM Tris, pH 7.5, 6% Ficoll PM-400, 0.2% Sarkosyl, 20 mM EDTA) using PDMS microfluidic devices (https://dropletkitchen.github.io/) according to the recommendations of (Macosko et al., 2015). Droplets were then broken using 1H,1H,2H,2H-perfluoro-1-octanol (Merck) and ChemGene beads collected by centrifugation and subjected to cDNA synthesis (Maxima H- RTase), introducing the molecule and cell barcode to every transcript from a single cell (termed a 'STAMP'). 1000 STAMPs from each sample were then selected for PCR amplification (15 cycles), library preparation (Nextera XT, Illumina) and Illumina sequencing by synthesis using a custom read 1 primer (NextSeq-500 platform; version 2 chemistry - high output setting; 20 bp read 1, 50 bp read 2 and an 8 bp index 1). Species-mixing experiments are routinely performed in our laboratory and have determined that our implementation of the Drop-Seq protocol robustly achieves single-cell encapsulation and captures transcriptomes from single-cells with high specificity (98.5% of cell encapsulation events are single-species; data not shown). Raw sequencing reads were converted to a sorted unmapped BAM file (FastqToSam, Picard bundled in Dropseq-tools v2.1) and filtered to remove all read-pairs with a barcode base quality of <10. The second read was trimmed at the 5' end to remove any TSO-adapter sequence and at the 3' end to remove polyA tails. Reads were aligned against human reference genome (HG38) using STAR aligner (v2.5.0a), then sorted/converted/merged to a BAM with

a tag “GE” onto reads for data extraction. The DigitalExpression program (Dropseq-tools v2.1) performed digital counting (DGE) of the mRNA transcripts (unique molecular identifiers to avoid double counting reads/PCR duplicates) and created a DGE matrix (one measurement per gene per cell).

DropSeq Analysis

The final matrixes contained 14,376, 20,260, 33,536 cell barcodes and 24,479, 25,359, 27,715 genes for the chemotherapy, CRT and chemo-naive datasets, respectively (351, 513 and 929 million data-points, respectively). A Seurat (Rahul Satija (NA). Seurat: R toolkit for single cell genomics. R package version 4.1.1.) object containing only those cells with a minimum of 150 and a maximum of 4000 genes was created. The percentage of mitochondrial RNA content in each cell was calculated and cells with more than 25% mitochondrial genes were removed. Genes associated with dissociation (van den Brink et al., 2017) were identified and cells whose transcriptome contained more than 10% of genes from this list were excluded from further analysis. A total of 38,676 cells expressing 57,095 genes remained containing more than 2.208 billion transcripts across the 3 data sets. Counts were normalized and variable genes selected using SCTransform with percentage mitochondrial content, cell cycle phase, gene count, sequencing batch and DropSeq run as latent variables to regress out with vars.to.regress. We then identified variable features (Seurat: ‘FindVariableFeatures’) using the ‘vst method’ on the SCT assay, prior to PCA (Seurat: ‘RunPCA’), SNN Graph construction (Seurat: ‘FindNeighbors’), cluster determination (Seurat: ‘FindClusters’) and Uniform Manifold Approximation and Projection (Seurat: ‘RunUMAP’). Cluster-specific genes were detected using the Wilcoxon rank sum test in (Seurat: FindAllMarkers) ($p < 1 \times 10^{-3}$ was considered significant). The annotations of cell identity for each cluster were defined by the expression of canonical markers and *a priori* knowledge (Supplementary Data). Our treatment naïve sample data was used as reference atlas for automated cell identification which was applied to the

chemotherapy and CRT treated datasets using canonical correlation analysis, cells with a prediction score < 0.8 were excluded from further analysis. A total of 16,904, 14,446 and 8,587 cells were retained for the chemo-naïve, CRT and chemotherapy treated cohort, respectively. After manual inspection of lineage markers of each dataset, further low-quality or doublet cells were removed, as well as patients with low cellularity (<250 cells/ patient: OAC1512 (n=51 cells) from the CRT dataset and OAC2610 (n=44 cells) and OAC1710 (n=231 cells) from the chemotherapy dataset) (**Figure S1B**). Next, the 3 datasets were merged and further manual inspection resulted in the removal of patient OAC36 (2,106 cells) due to mainly consisting of low quality/ doublet cell barcodes (Figure S1B).

Following our first pass clustering analysis and cell type classification broadly classified cell data subsets of Immune cells, Cancer Cells and Stromal tissue were created and re-analysed (‘FindVariableFeatures’, ‘RunPCA’, ‘FindNeighbors’, ‘FindClusters’, ‘RunUMAP’). For each cell subset Seurat’s ‘FindAllMarkers’ (default settings Wilcoxon Rank Test) and the ‘AverageExpression’ function was used to explore and classify clusters by their mean expression of canonical markers for each cell subset.

We carried out further quality control after sub-setting and removed OAC2210 from the Cancer dataset due to low cell numbers (n=3) (**Figure S1B**). Module scoring was applied to the cancer cells using The MSigDB Hallmark collection (Liberzon et al., 2015; Subramanian et al., 2005) and Seurat’s “AddModuleScore” function. Enrichment of cell types and subtypes was calculated 2x2 contingency table based X^2 tests.

The Stromal tissue subset (16,713 cells) were subclustered into “Endothelial” cells (n=1,619 cells), “Pericyte and Smooth Muscle” cells (n=845 cells), “Squamous” cells (n=5,525), “Fibroblasts”(n=8,468 cells) and “Quiescent” cells (n=256 cells). After our first pass clustering analysis, removal of cells that were low-quality/uninformative, or likely contaminated by other cell types (doublets) left 6,415 (76%) fibroblasts, 1,427(88%) endothelial cells, 809 (96%)

pericytes/smooth muscle cells, and 5,146 (93%) squamous cells. Quiescent cells were not analysed further due to the low number of cells present in the cluster. The Fibroblasts were analysed as a complete group and then the tumor derived fibroblasts were analysed as a "CAF" subcluster of 2,970 cells. The finalised datasets were then re-clustered and analysed. We created an immune cell data subset by selecting cells identified as: B cells, plasma cells, macrophages, mast cells and T-cells. The immune cell dataset consisted of 18,229 cells (1,604 normal-derived cells and 16625 tumor-derived cells) and was used to calculate the broad immune cell counts and proportions. For our secondary cell filtering step, we created separate cell datasets for B cells, plasma cells, macrophages, T cells and mast cells. For each separate cell dataset, we iteratively pruned cells where we were uncertain of their cell identity (by marker log-normalized expression < 1) or to remove the low-quality/uninformative cell captures retained following our first pass clustering analysis using the steps described above. We retained 3475/5950 (58%), 7828/10678 (73%), 1869/2640 (71%) and 1239/1325 (94%) cells from the B cells, T cells, macrophages and mast cell datasets, respectively. The pruned datasets were then re-clustered using the steps described above prior further analyses as described in the manuscript.

Inferred copy number variation (InferCNV) analysis

Copy number variation in malignant cells was performed using InferCNV (Puram et al., 2017; Tirosh et al., 2016a, 2016b). Cells identified in the Seurat object as malignant were included only if there were at least 30 cells from that patient sample leaving 7, 5 and 12 patient samples from the chemo, CRT and chemo-naïve datasets respectively, for analysis. Samples retained for analysis were compared to a reference set of "normal" cells from the same samples using InferCNV with default settings except cutoff = 0.1, HMM = TRUE.

(Trapnell et al., 2014)

Trajectory analysis using CytoTRACE

Webtool CytoTRACE (cellular trajectory reconstruction analysis using counts and expression) (Gulati *et al.*, 2020)located at https://cytotrace.stanford.edu/ was used to infer cell trajectory of fibroblast cell populations. The read counts matrix and the phenotype data (CAF/NOF type) for the fibroblasts subset was used as the input for the analysis and results downloaded from resulting output.

COTAN

R package COTAN (co-expression tables analysis) (Galfre *et al.*, 2021) version 1.2.0 was used to infer gene co-expression in single cells of the fibroblast population. Default parameters were used and workflow at http://bioconductor.org/packages/release/bioc/vignettes/COTAN/inst/doc/Guided_tutorial.html was followed. Clustering based on COTAN co-expression scores was performed using the workflow at https://seriph78.github.io/Cotan_paper/Gene_clustering.html using the two of the top differentially expressed genes for each CAF cluster: CAF1 – *COL1A1*, *COL3A1*; CAF2 – *CXCL8*, *CXCL1*; CAF3 – *PI16*, *GSN*; CAF4 – *PLA2G2A*, *SFRP2*; CAF5 – *POSTN*, *F3*. The parameter for number of associated genes per marker (n.genes.for.marker) was set at 20 and an optimum number of 10 clusters was obtained using the PAM clustering algorithm. Genes from the clusters containing GSN and POSTN were used for pathway analysis and cell type signature identification in metascape (Zhou et al., 2019).

SCENIC

R package SCENIC (single-cell regulatory network inference and clustering) [https://doi.org/10.1038/nmeth.4463] version 1.0 was used to analyse transcription factor (TF) activity in fibroblasts. Default parameters were used as set out in the SCENIC workflow at https://github.com/aertslab/SCENIC/blob/master/inst/doc/SCENIC_Running.html to obtain regulon scores (score based on expression of genes in the gene regulatory network for a TF) and binary status (on/off) for each transcription included in the analysis. Regulon score for

each cell was obtained from 3.4_regulonAUC.Rds and binary status was obtained from 4.1_binaryRegulonActivity.Rds and mapped onto the fibroblast UMAP.

CellPhone DB, CrossTalkR and NicheNet

Using the CellPhoneDB (version 2.1.3; Custom database, method settings = 1000 iterations, 0.1 threshold and p-value = 0.05) significant means output as input for CrossTalkR analysis (version 1.3.0; recommended settings) enabled us to determine the differential Cell-Cell interaction networks (CCI) and Cell-Gene Interactions (CGI) present and utilize network topological measures to highlight CCIs or CGIs that can be ranked for (1) importance (PageRank), and (2) scored based on its role as an influencer (out-going edges), listener (in-goings edges) or mediator (betweenness centrality). NicheNet analysis was performed using NicheNet v2 according to Sang-aram et al., Nature Protocols (2025) (https://www.nature.com/articles/s41596-024-01121-9#citeas). For the sender-focused (CAF1, 2, 3, 4, or 5 subtypes) and sender-agonistic analyses normal tissue-derived cell captures were excluded and TRG3 cases were included into the non-responders' group to ensure sufficient sampling for all cell lineages. Differential expression analysis was performed using Seurat's Wilcoxon test, with the following cut-offs set: minimum % expression = 10%, log2FoldChange = 0.25, geneset = DE (all DE genes between responders and non-responders) and adjusted p-value = 0.05. The top 30 ligands (ligands with the highest area under the precision-recall curve, AUPR) from each sender-focused were manually inspected and cross-referenced against any predicted target-gene expression changes in the receiver cell lineages (Antigen presenting cells, B lymphocytes, Cancer cells, endothelium, T lymphocytes). We used the sender-agnostic NicheNets to compare the rankings of the sender-focused NicheNets, here highly ranked ligands (within the top 20 were visualized) were considered to have the highest likelihood of importance in the cell-cell communication networks. To prioritize the top ligand-receptor pairs (LR) across multiple receiver cell types we concatenated all sender-receiver cell

type tables and created a final prioritization table for each receiver cell type retaining the top 10 LRs for each. Excluding CAF subtype-T lymphocyte interactions, we reported predicted target genes for only the top two or three CAF subtype interactions with APCs and endothelial cells. Importantly, these interactions retained importance as they were also ranked highly in in the sender-agnostic NicheNets (within the top 10 prioritized ligands from that analysis). Random forest models were not performed as the CAF have a high variability between cases. Prior human models from the NicheNet v2 authors were sourced from (https://zendo.org/record/7074291/files) and used in all our analyses.

Single Cell RNA-seq Validation Cohorts

Count matrices for fibroblasts of six validation cohorts were obtained from their individual studies: head and neck cancer (Puram *et al.*, 2017); oesophageal squamous cell carcinoma (Zhang et al., 2021); breast cancer (Qian *et al.*, 2020); obtained from https://lambrechtslab.sites.vib.be/en/pan-cancer-blueprint-tumour-microenvironment-0]; gastric cancer (Kim et al., 2022); pancreatic adenocarcinoma (Raghavan et al., 2021); and colorectal cancer (Qian *et al.*, 2020), obtained from https://lambrechtslab.sites.vib.be/en/pan-cancer-blueprint-tumour-microenvironment-0]. Seurat objects were created for all datasets and any non-fibroblast cell removed. Fibroblasts were separated into three categories based on scores for the myofibroblast and universal signatures used in this study – Myofibroblast (high myofibroblast, low universal score); Universal (low myofibroblast, high universal score); Other (low/medium myofibroblast, low/medium universal score). Median and interquartile values for GSN were calculated and compared between myofibroblasts and universal fibroblasts. The Wilcoxon rank-sum test was used to test differences between fibroblast populations for significance.

Enrichment and Gene Set Variation Analysis (GSVA)

The MSigDB (MSigDB_Hallmark_2020) hallmark collection (Liberzon *et al.*, 2015) (Subramanian et al., 2005) was selected as the gene list source for the pathway activity analysis using the ErichR R-based tool (Chen et al., 2013; Kuleshov et al., 2016). Where DEGs with P value <0.05 and a logFC >0.25 or the gene list for each cluster of co-expressed DEGs across the cancer cell clusters were used as input for enrichment analysis. The Bioconductor package GSVA (Hanzelmann et al., 2013) was performed using the canonicalC2BroadSets (c2.all.v7.5.1), filtered for KEGG, REACTOME and BIOCARTA sets on the significant DEGS (P<0.05) identified using limma (Smyth, 2004) on the log(cpm) normalized counts.

TME profiling in bulk tumors

RNA-seq FPKM values were obtained for 174 esophageal adenocarcinoma bulk samples from OCCAMS and 87 bulk samples from TCGA, respectively. The composition of the tumor microenvironment was assessed based on expression profiles through Gene Set Variation Analysis (GSVA) (Hänzelmann et al., 2013) via the GSVA package in R using the cell type-specific signatures derived from the single cell analysis (available on request).

Pseudobulk Analysis

Pseudobulking of the cancer single cell transcriptomics was performed using the Aggregated Feature Expression function (AggregateExpression()) in Seurat by the identity class('orig.patient') and restricted to features with greater than or equal to 50 counts in the Seurat object 'counts' slot. Cancer expression program/module scores were calculated on the single cell transcriptome data prior to aggregation using the mean score value for the identity class ('orig.patient').

Survival analysis

Survival analysis was performed using Kaplan-Meier analysis (using the Custom KMplotter tool (https://kmplot.com/analysis/index.php?p=service&cancer=custom_plot) to identify best performing expression cutoff and visualize KM plots) (Lanczky and Gyorffy, 2021) and

univariate and multivariate modelling was performed using Cox Proportional Hazards via the R Packages, survival() and finalfit() on overall survival data from TCGA EAC cohort (data release: 36) and OCCAMs cohorts. Variables included in the multivariate analysis were, age, gender, stage(I-IV), and dichotomized gene expression or weighted expression score and only cases with complete data for all covariates were included. The 4-gene weighted expression score was calculated from RPKM/FPKM values, accordingly: (1**POSTN*+1**ZEB2*+1**ATF1*-2**GSN*)/4 and the alpha level was set at 5% ($p<0.05$) for all statistical tests (where < or > 61st percentile was associated with the highest HR value and significance). The 4-gene signature included, *GSN* as a positive marker for universal fibroblast presence in tumors, *ATF1* as a canonical regulator of GSN expression, *ZEB2* as a marker of EMT activity in EAC cancer cells and *POSTN* as a positive marker for myoCAF presence in EAC tumors. The selection of these genes was consistent with our hypothesis, *'EAC cancer cells undergoing EMT promote myofibroblast differentiation'*. We tested genes individually and selected the weighted average gene expression of all 4 genes (equation shown above) with the lowest false discovery rate using Cox Proportional Hazards (CPH) analysis (FDR: 15; P-values 1.2e-5, expression cutoff =-3.34; CPH assumption passed). Under independent multivariate modeling in the discovery cohort (TCGA EAC cases, n=82) only the 4-gene signature retained prognostic significance (Concordance = 0.72, S.E.-0.043; Likelihood ratio test = 29.2, p=6e-5; Wald test =26.1, p=2e-4 and Score (logrank) test=32.02, p=2e-05). Individual genes tested in a separate multivariate model did not retain independent prognostic significance when included with following co-variates (age, gender and stage). Cox.zph() function and Schoenfeld tests were used to test the proportional hazards assumption of the Cox Regression for all co-variates; none of which were identified as time-dependent variables over the OS follow up period. Bootstrapping (n=200) and cross-validation (n=10 number of groups of omitted observations) in the TCGA cases revealed Dxy/2+0.5 values (0.69 and 0.70, respectively) similar to the model C-index

(Concordance) of 0.728. In the OCCAMs cohort the model (4-gene signature cut-off = -0.71; FDR =20%) achieved a C-index of 0.65 and passed the Cox.zph() function and Schoenfeld tests and returned equivalent Dxy/2+0.5 values when Boot-strapped (0.60) and cross-validated (0.58) in comparison to the C-index.

Immunohistochemistry

Optimization and immunohistochemical staining of the cohort were performed on 4um thick formalin-fixed paraffin embedded tissue sections. Appropriate heat-induced epitope retrieval for each stain was performed on a Dako PT Link instrument using Dako FLEX TRS Low pH or Dako FLEX TRS High pH retrieval buffers. Staining was performed individually for CD3 (Ready-to-Use formulation with Dako FLEX TRS High pH retrieval; IS503, Dako), EpCAM (1:60 dilution with Dako FLEX TRS Low pH retrieval; Dako), POSTN (1:500 dilution with Dako FLEX TRS Low pH retrieval; ab14041, abcam), α-SMA (1:200 dilution with Dako FLEX TRS High pH retrieval; M085129-2, Dako), and GSN (1:500 dilution with Dako FLEX TRS High pH retrieval; 12953S, Cell Signaling Technologies) using a Dako Autostainer 48S Link automated staining machine according to the manufacturer's instructions. A separate section adjacent to these stains was taken to perform routine hematoxylin & eosin staining to assist in identifying regions containing tumor and normal esophageal and gastric epithelium.

Digital pathology-based quantification of immunohistochemistry

Immunohistochemical stains were quantified using a combined digital pathology and expert pathologist approach. Whole slides were scanned using a digital slide scanner Zeiss Axioscan.Z1. Quantification of staining was conducted on the digital slide images using QuPath (version 0.3.2) (Bankhead et al., 2017). Automated segmentation of tissue sections was performed on whole slide images and corrected manually to ensure accuracy. A certified pathologist (R.R.) marked up the H&E-stained slide image in QuPath to determine areas of tissue containing tumor and normal esophageal or gastric epithelium. These labels were

transferred to adjacent sections bearing IHC stains and corrected for each section by a non-pathologist scientist (B.P.S.), with annotations confirmed by the pathologist. CD3 stains were quantified using a previously established method (Sharpe *et al.*, 2022), where the positive cell detection tool in QuPath was used to count CD3+ cells, with outputs measured in frequency of CD3+ cells per mm^2 of tissue. EpCAM, POSTN, GSN and α-SMA were quantified by thresholding of deconvolved DAB optical density to determine the area of tissue stained with the antibodies, as previously established (Loughrey et al., 2018). A percentage of total tissue area, tumor area, and normal epithelial area was determined for each by dividing the DAB thresholded area by the total area of each annotation and multiplying by 100. Groovy scripts for tissue detection and all downstream image analysis are available online at Zenodo.

Cell culture experiments

Human Fetal Foreskin Fibroblast 2 (HFFF2) wells were obtained from ECACC (Cat no: 86031405). Cells were maintained in Dulbecco's modified Eagle's medium (DMEM, Invitrogen) supplemented with 10% (v/v) fetal calf serum (FCS, Autogen Bioclear), 2 mM L-glutamine and 1% penicillin/streptomycin (Invitrogen) at 37 °C and 5% CO2. TGF-β was used to induce myofibroblast differentiation and was manufactured in-house by Dr Patrick Duriez (Centre for Cancer Immunology, University of Southampton, UK).

Fibroblast differentiation

HFFF2 cells were grown under cell culture conditions for 6h. After the cells adhered, media was then changed to low serum (1%) DMEM for 24h before the addition of 2 ng/mL TGF-β or vehicle (4 mM HCl, 0.4 mg/mL BSA) and grown for 72h (time points: 6h, 24h, 48h and 72h).

Quantitative real-time PCR

Extracted RNA of the TGF-β treated or vehicle HFFF2 cells was subjected to cDNA synthesis using the High-Capacity Reverse Transcription Kit (Applied Biosystems). The TaqMan assays to *POSTN, ACTA2, ZEB1, ZEB2, ATF1* and *GSN* were run in biological triplicate on the Real-

time PCR system (Thermo Scientific), according to the manufacturer's instructions with a VIC-TAMRA-labeled β-actin assay (Thermo Scientific) as an internal control. Quantities of RNA per well were interpolated from a standard curve, normalized to the internal control, and then normalized to control samples as indicated.

Western Blotting

Protein expression of POSTN, ACTA2, ZEB1, ZEB2, ATF1 and GSN was carried out by Western Blotting in biological triplicates. Protein was extracted using cell lysis buffer (Cell Signaling Technology). Proteins were resolved on 3–8% Bis-Tris acrylamide gels and transferred onto PVDF membranes using the X-Cell II and iBlot systems according to manufacturer's instructions (Invitrogen). Blocking and antibody incubations were done in 3% low-fat milk in PBS–0.025% Tween 20, and washes were in PBS–0.1% Tween 20. Images were collected using a CCD camera.

QUANTIFICATION AND STATISTICAL ANALYSIS

Statistical Analysis

Statistical analysis was performed using R version 4.1.1 (One Push-Up). Categorical variables were compared using the Chi-Squared test. A two-tailed Student's t test was used to analyze parametric data and non-parametric data was analyzed by a Mann-Whitney test (unpaired data) or Wilcoxon test (paired data). To assess survival differences, Kaplan-Meier curves were produced and analyzed by log-rank testing. p values <0.05 were regarded as statistically significant. Unless stated otherwise, p values are represented as follows, *p<0.05, **p<0.01, ***p<0.001 and ****p<0.0001.

SUPPLEMENTAL INFORMATION TITLES AND LEGENDS

Supplementary Figure Legends

Figure S1. Study overview (A) Overview of EAC patient cohort across the three treatment groups **(B)** Overview of quality control methods during bioinformatics analysis

Figure S2. Fibroblast gene expression following treatment. (A) Fold change bar chart of EAC TME components comparing NAT-treated samples to surgery only treatment-naïve samples. FDR = False Discovery Rate (Red dotted line). **(B-E)** Volcano plots of differential gene expression, grouped as indicated (Blue and red dots), using an adjusted p-value (Benjamini-Hochberg) of 0.05 and a log2-fold change cut off set to 0.25.

Figure S3. Stacked Violin Plots of marker gene expression in CAFs from Croft et al. Violins are split by normal vs tumor status and colored boxes highlight the conserved cell phenotypes across the Walker et al and Croft et al data sets.

Figure S4: Treatment response and the EAC TME with focus on CAF dynamics during neo-adjuvant treatment. (A) UMAP of cell lineages, patient ID and tumor microenvironment component. **(B)** UMAP of cell source stratified by treatment status (T = tumor (red dots), N= normal (blue dots)). Black arrows indicate the cancer-associated fibroblast cell clusters. NOF clusters are excluded for better visualization **(C)** UMAP of CAF clusters stratified by treatment status. **(D)** Stacked bar charts of CAF subtype proportions in 9 patients (with greater than 50 CAF cells sampled). Grouped by treatment type and Mandard tumor regression grade indicated.

Figure S5: Cell communication analysis of OAC tumors following neoadjuvant therapy. (A) Venn-diagram of predicted ligand – receptor interactions by CellPhoneDB in CAF3 cells from responders (CAF3_R) and non-responders (CAF3_NR) to NAT. The top three enriched gene ontology terms and associated genes are indicated in the tables (EnrichR:

https://maayanlab.cloud/Enrichr/). **(B)** Sankey plots listing predicted cell source, ligand - receptor and cell receiver interactions associated with NAT response. LRscores are indicated by the divergent color scale. NR = Non-responders, R = Responders. **(C)** Comparative Cell-Cell Interaction network in non-responders versus responders for each treatment type (P-value filter set at 0.2), where node size (Page rank odds-ratio) and edge thickness (% of interactions) represents importance of the cell communication between cells. The arrows indicate the signal direction and colour the activation status (Brown = Up & Blue = Down).

Figure S6: Gelsolin expression patterns across EAC tissue specimens. (A) Histologically normal esophageal stratified squamous epithelium with scattered GSN+ cells in the basal and suprabasal epithelial layers **(B)** EAC with heterogeneous membranous and cytoplasmic positive GSN expression in tumor epithelium **(C)** Lymphoid aggregate within EAC showing membranous and cytoplasmic positive GSN expression in scattered cells. **(D)** EAC with negative GSN expression in tumor epithelium, and positive GSN expression in stromal fibroblasts surrounding it **(E)** EAC with negative GSN expression in tumor epithelium, positive GSN expression in stromal fibroblasts, and GSN positive cells infiltrating within tumor epithelium **(F)** EAC with negative GSN expression in tumor epithelium and positive GSN expression in cells localized to intraepithelial lumina. Scale bars - 250um.

Supplementary Table Titles

Table S1. Clinical and pathological characteristics of the primary tumor series

Table S2. Clinical characteristics for each EAC patient.

Table S3: Prognostic significance of the weighted gene expression model in TCGA pan-cancer datasets.

Table S1: Clinical and pathological characteristics of the primary tumor series

	Treatment naïve EAC patients (n=13 samples)	Chemoradiotherapy treated EAC patients (n=6 samples)	Chemotherapy treated EAC patients (n=9 samples)
**Age in years (range)***	70.8 (46- 82)	64.5 (51- 76)	65.4 (43- 79)
**Gender (N; %)**			
Males	10 (76.9)	4 (66.7)	8 (88.9)
Females	3 (23.1)	2 (33.3)	1 (11.1)
**Tumour location (N; %)**			
Distal oesophagus	7 (53.8)	5 (83.3)	5 (55.6)
Gastro-oesophageal junction	6 (46.2)	1 (16.7)	4 (44.4)
**cT Stage (N; %)**			
2	5 (38.5)	0 (0)	1 (11.1)
3	4 (30.8)	4 (66.7)	5 (55.6)
4	4 (30.8)	2 (33.3)	2 (22.2)
4a	0 (0)	0 (0)	1 (11.1)
**cN Stage (N; %)**			
0	5 (38.5)	0 (0)	0 (0)
1	7 (53.8)	4 (66.7)	4 (44.4)
2	1 (7.7)	2 (33.3)	5 (55.6)
**cM Stage (N; %)**			
0	13 (100)	6 (100)	9 (100)
**pT Stage (N; %)**			
0	1 (7.7)	1 (16.7)	1 (11.1)
1a	0 (0)	0 (0)	1 (11.1)
1b	3 (23.1)	0 (0)	0 (0)
2	0 (0)	2 (33.3)	1 (11.1)
3	7 (53.8)	3 (50.0)	4 (44.4)
4a	1 (7.7)	0 (0)	1 (11.1)
Staging Lap only	1 (7.7)	0 (0)	0 (0)
**pN Stage (N; %)**			
0	5 (38.5)	4 (66.7)	4 (44.4)
1	3 (23.1)	1 (16.7)	2 (22.2)
2	1 (7.7)	0 (0)	0 (0)
3	2 (15.4)	1 (16.7)	3 (33.3)
3a	1 (7.7)	0 (0)	0 (0)
Staging Lap only	1 (7.7)	0 (0)	0 (0)
**pM Stage (N; %)**			
0	10 (76.96)	5 (83.3)	9 (100)
1	2 (15.4)	1 (16.7)	0 (0)
Staging Lap only	1 (7.7)	0 (0)	0 (0)
**Neoadjuvant treatment (N; %)**			
CAPOX	0 (0)	0 (0)	3 (33.3)
CROSS	0 (0)	6 (100)	0 (0)
ECX	0 (0)	0 (0)	1 (11.1)
FLOT	0 (0)	0 (0)	5 (55.5)***

No neoadjuvant treatment	13 (100)**	0 (0)	0 (0)
**Response to neoadjuvant therapy (N; %)$**			
TRG1	0 (0)	1 (16.7)	1 (11.1)
TRG2	0 (0)	1 (16.7)	1 (11.1)
TRG3	0 (0)	1 (16.7)	4 (44.4)
TRG4	0 (0)	3 (50.0)	1 (11.1)
TRG5	0 (0)	0 (0)	2 (22.2)
No neoadjuvant	13 (100)	0 (0)	0 (0)

CAPOX: capecitabine and oxaliplatin; CROSS: carboplatin paclitaxel followed by radiation of 41.4 grys; ECX: epirubicin cisplatin and capecitabine; FLOT: 5-fluorouracil leucovorin oxaliplatin and docetaxel.
**Values indicate Median (range).*
***Two patients in this cohort have matched naïve and treatment samples: ID OAC22 of the chemo naïve group underwent 2 cycles of durvalumab followed by CRT treatment and ID OAC23 of the chemo naïve group underwent CRT treatment only.*
****One patient in this cohort, ID OAC37, received 2 cycles of durvalumab followed by chemotherapy treatment*
$TRG is a scale from 1-5 where 1 = complete absence of residual cancer cells and 5 = continued growth of cancer cells with no evidence of regression in response to cytotoxic treatment

Table S2: Clinical characteristics for each EAC patient (n=26 patients, n=28 samples)

	ID	Sex	Age	Tumour location	cT Stage	cN Stage	cM Stage	pT Stage	pN Stage	pM Stage	Pre-Op Treatment	Response to neoadjuvant therapy[†]	IHC Cluster[$]
Treatment-naïve	OAC132	M	69	DO	2	0	0	1b	0	0	**	**	1
	OAC55B	F	69	GOJ	4	1	0	3	1	1	**	**	1
	OAC2811	M	82	GOJ	3	1	0	4a	3a	1	**	**	2
	OAC1212	M	78	DO	3	2	0	3	3	0	**	**	2
	OAC233	M	65	DO	2	0	0	3	1	0	**	**	2
	OAC67	M	78	DO	3	1	0	3	1	0	**	**	1
	OAC26	M	66	GOJ	2	0	0	3	3	0	**	**	2
	OAC31	M	46	GOJ	2	1	0	1b	0	0	**	**	1
	OAC32	M	82	GOJ	3	1	0	3	2	0	**	**	2
	OAC42	M	78	DO	2	0	0	1b	0	0	**	**	1
	OAC276	F	66	GOJ	4	0	0	*	*	*	**	**	N/A
	OAC22 (OAC33)	F	65	DO	4	1	0	3	0	0	**	**	N/A
	OAC23 (OAC34)	M	76	DO	4	1	0	0	0	0	**	**	N/A
Chemoradio-therapy treated	OAC1411	F	61	DO	3	2	0	3	1	0	CROSS	TRG4	2
	OAC174	M	72	DO	3	1	0	2	0	0	CROSS	TRG4	1
	OAC33	F	65	DO	4	1	0	3	0	0	CROSS	TRG4	2
	OAC34	M	76	GOJ	4	1	0	0	0	0	CROSS	TRG1	N/A
	OAC35	M	62	DO	3	1	0	3	3	1	CROSS	TRG2	2
	OAC36	M	51	DO	3	2	0	2	0	0	CROSS	TRG3	1
Ch e m	OAC1210	M	48	DO	4	2	0	3	1	0	FLOT	TRG2	N/A

	OAC2210	M	79	GOJ	4a	1	0	1a	0	0	FLOT	TRG3	N/A
	OAC263	M	74	GOJ	3	1	0	0	0	0	CAPOX	TRG1	N/A
	OAC29	M	70	GOJ	3	2	0	1b	0	0	FLOT	TRG3	1
	OAC3010	M	74	DO	3	2	0	4a	3	0	CAPOX	TRG5	1
	OAC307	M	72	DO	2	1	0	2	1	0	FLOT	TRG3	N/A
	OAC37	M	43	DO	3	1	0	3	3	0	FLOT	TRG5	2
	OAC44	F	69	GOJ	3	2	0	3	3	0	ECX	TRG3	N/A
	OAC46	M	60	DO	4	2	0	3	0	0	CAPOX	TRG4	1

*Footnote: DO: distal oesophagus; GOJ: gastric oesophageal junction; CAPOX: capecitabine and oxaliplatin; CROSS: carboplatin paclitaxel followed by radiation of 41.4 grys; ECX: epirubicin cisplatin and capecitabine; ECX: epirubicin cisplatin and capecitabine; FLOT: 5-fluorouracil leucovorin oxaliplatin and docetaxel. * Patient was sampled at pre-treatment biopsy (staging lap) and did not undergo resection. **No neoadjuvant treatment. [†]Response to therapy assessed by Mandard Tumour Regression Grade. TRG1 = no residual cancer cells, TRG2 = Few cancer cells, TRG3 = fibrosis outgrows cancer cells, TRG4 = Cancer cells outgrow fibrosis and TRG5 = Absence of regressive changes. OAC22 and OAC33 are the same patients at different timepoints; OAC23 and OAC34 are the same patients at different timepoints. [$]IHC Cluster 1 defines patients with a low POSTN/αSMA staining by IHC, IHC Cluster 2 defines patients with a high POSTN/αSMA staining by IHC (data available for 19 patients).*

Table S3: Prognostic significance of the 2-gene expression signature in TCGA pan-cancer datasets

Cancer Type[1,2]	Sample number (N)	HR (95% CI)	P-value
Kidney Renal papillary cell carcinoma	193	3.63 (1.56 – 8.42)	0.0013
Cervical squamous cell carcinoma	203	2.77 (1.45 – 5.27)	0.0013
Uterine corpus endometrial carcinoma	363	2.11 (1.24 – 3.59)	0.0049
Sarcoma	173	1.82 (1.11 – 2.99)	0.017
Kidney Renal clear cell carcinoma	355	1.7 (1.14 – 2.54)	0.0087
Lung adenocarcinoma	337	1.63 (1.11 – 2.37)	0.011

[1](Data from Pan-cancer KMPlotter, accessed May 2024)
[2]*Bladder Cancer, Breast Cancer, Esophageal Squamous Cell Carcinoma, Liver hepatocellular carcinoma, Lung squamous cell carcinoma, Head and Neck Squamous Cell Carcinoma, Ovarian cancer, Pancreatic ductal adenocarcinoma, Pheochromocytoma and Paraganglioma, Rectum adenocarcinoma, Testicular Germ Cell Tumor, Thymoma, Stomach adenocarcinoma and Thyroid carcinoma were not significant for this gene signature*
